# Unraveling the Design Principle for Motif Organization in Signaling Networks

**DOI:** 10.1371/journal.pone.0028606

**Published:** 2011-12-02

**Authors:** Samrat Chatterjee, Dhiraj Kumar

**Affiliations:** Immunology Group, International Centre for Genetic Engineering and Biotechnology, New Delhi, India; Michigan State University, United States of America

## Abstract

Cellular signaling networks display complex architecture. Defining the design principle of this architecture is crucial for our understanding of various biological processes. Using a mathematical model for three-node feed-forward loops, we identify that the organization of motifs in specific manner within the network serves as an important regulator of signal processing. Further, incorporating a systemic stochastic perturbation to the model we could propose a possible design principle, for higher-order organization of motifs into larger networks in order to achieve specific biological output. The design principle was then verified in a large, complex human cancer signaling network. Further analysis permitted us to classify signaling nodes of the network into robust and vulnerable nodes as a result of higher order motif organization. We show that distribution of these nodes within the network at strategic locations then provides for the range of features displayed by the signaling network.

## Introduction

The sensitivity of intracellular signaling systems to variations in input stimuli is evident from the multiple outputs that are produced at the level of changes in gene expression and cellular activities [1]–[7]. However, such systems must also guard against the generation of inappropriate and non-specific responses that may potentially be induced through either noise in the external milieu, or through stochastic perturbations of the intracellular components (e.g. mutations, alterations in protein turn-over rates etc). An understanding of how this fine balance between robustness and sensitivity is achieved is, however, currently lacking.

The dynamic properties of signaling networks are defined by the motifs that are embedded in them [8]–[11]. A variety of motifs have been discovered and these collectively constitute the building blocks of biological networks [12]–[14]. Recent studies have shown that the pattern of motif organization defines the information processing capabilities of the signaling network, influencing the specificity and plasticity of input/output relationships [12], [15]. Thus, for example, a complex motif organization is associated even with a simple three-tiered cascade such as the MAP kinase pathway [16]. This motif organization is critical for defining signaling responses of the pathway to variations in both the nature and strength of the input signal. Importantly, this motif organization also explains the tissue-specific variations in the ERK response to input stimuli [16]. A more integrated perspective was provided by studies in hippocampal CA1 neurons, which revealed that a variety of regulatory motifs were formed downstream to signaling cascades and described that key regulators of plasticity were highly connected nodes required for the formation of regulatory motifs [17]. These and other such related findings, therefore, led us to speculate whether the pattern of motif organization in a signaling network also contributes towards defining its robustness versus adaptability/sensitivity threshold.

Of the various signaling motifs described in the literature those belonging to the class of Feed forward loops (FFLs) are the most versatile, being endowed with properties like imparting persistence, delay or inhibition in signal output depending on the signs of each of the links in the motif [13], [17]. Further, motifs are considered as the building blocks of the signaling networks and accordingly we have recently shown integration of FFLs forming larger regulatory modules, with a consequent amplification of the regulatory features [16]. Therefore, we opted to examine the higher order organization of three-node FFLs in signaling networks, to determine their effects on the signal processing and cellular response. To probe the issue of maintenance of response-specificity, we selected a condition wherein cells were subjected to varying levels of stochastic perturbations and evaluated the consequent steady state value, rather than the kinetic features, of the output attained [3], [11], [15], [16], [18]–[25]. It is the change in steady state levels that have been shown to govern the outcome in complex biological processes such as adaptability, immune memory, development, and cell differentiation [15], [25], [26].

## Results

### A mathematical model for output from 3-node FFLs

**Figure 1:**
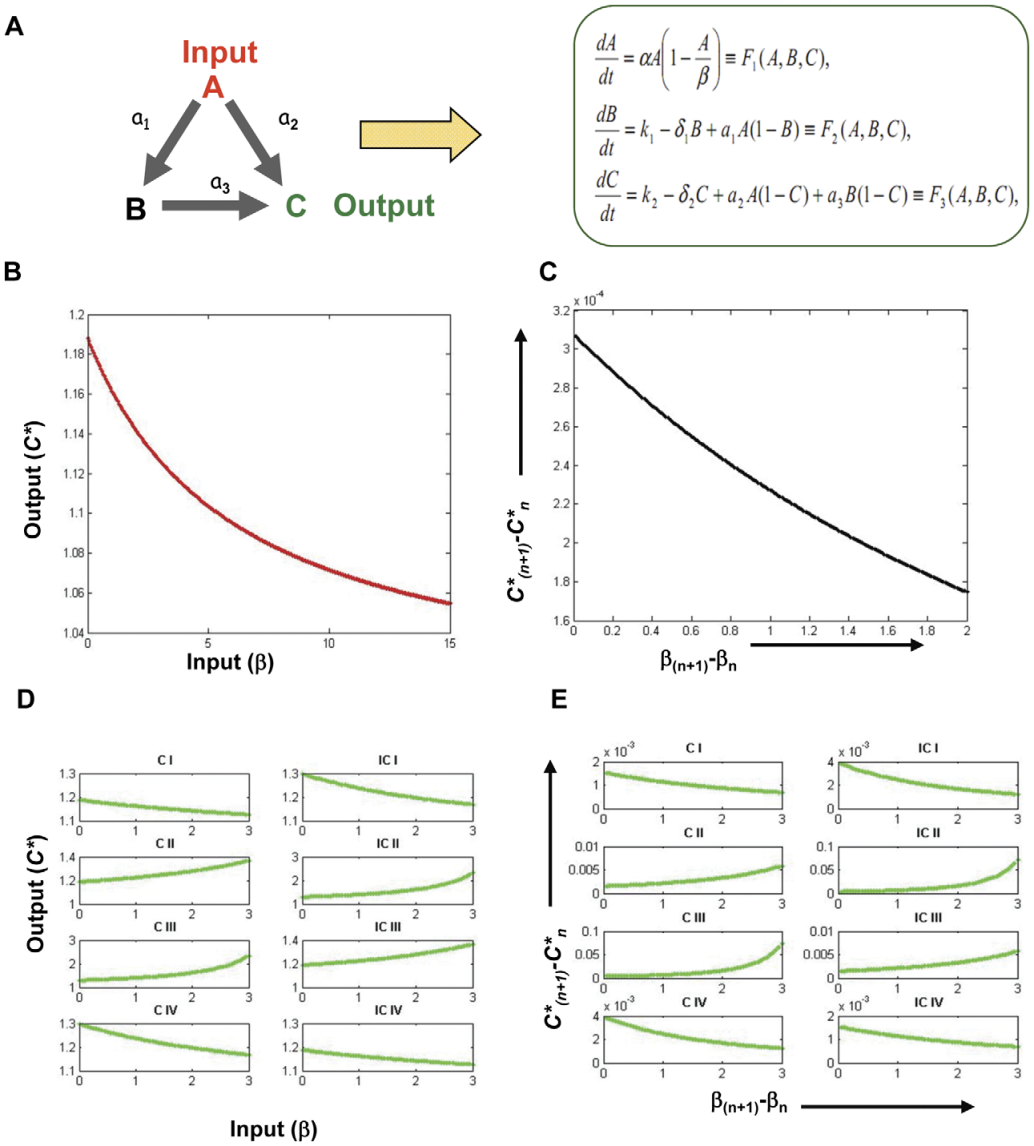
A mathematical model of three node FFLs. The typical feed-forward loop of three nodes is represented in panel A, along with the model. Parameters 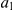, 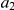 and 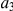 shows the relationship between the three nodes as depicted. It is important to note that by changing signs of 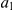, 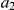 and 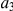 eight different motif architectures could be obtained. The specific input/output relationship is shown in Panel B, where output at node 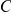 is plotted against the input 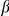. Panel C shows unit change in output (O_1_-O_1_) as a function of per unit change in input 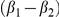. Plots corresponding to panels C and D for each of the eight possible FFLs (see text) are shown in panel D and E. A motif specific I/O relationship is evident in the figure.

We started with a simple model for a feed forward loop consisting of three nodes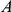, 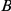 and 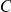 (Fig. 1A). Node 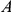 receives the input signal which then influences the output node 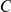 either directly or via additional regulatory node 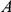. These relationships represent signaling events in a cell, either immediately downstream to some receptor or intermediate signaling events several steps following the initial receptor activation. Response of a biological system depends largely on how information about an environmental change or stimuli is relayed to the nucleus. Therefore cell signaling regulates most of the cellular responses [1], [27]. However with a limited number of molecules available to intercept, process and transmit the signal, it remains obscure how different biological information could be processed so precisely with nearly overlapping set of molecular agents [3], [7]. The three-node feed forward loop in this study was taken to represent signaling interactions between the molecules and how the nature of interaction might lead to specificity in cellular responses. Parameters governing the relationship between the nodes are 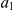, 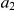 and 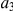 (Fig. 1A), and by simply changing their signs, eight different three-node FFL motif architectures could be described. Individual nodes were modeled for their activated form by defining their basal activation value as 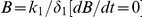 and 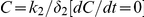. For each of the nodes, their respective inactive form was represented by deducting activated form from the unity thereby maintaining the overall amount of a given molecule constant [13], [15]. The steady state (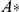) achieved by 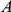 in response to a signal was equal to 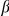 and was considered as our input to the motif. Enzymatic reactions were modeled on the basis of the law of mass action and the output was defined in terms of the steady state values achieved by node 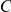
(denoted as 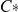).

In an FFL where all three edges exhibited positive regulation, an increase in input signal 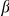 resulted in a corresponding increase in 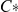
(Fig. 1B). To obtain more precise input/output (I/O) relationships, however, we calculated unit change in 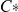 as a function of the change in input 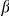. Interestingly, here we instead observed decrease in the increment in the output for a unit increase in input signal caused mainly due to the saturation effect (Fig. 1C). This profile, with its characteristic pattern of change in 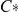, was taken as a descriptor of the response from the FFL that was specific to the input signal. By this approach the I/O relationships for all eight possible FFLs were examined. As shown in Fig. 1D, the response pattern generated from each motif was unique wherein many instances displayed a reduced value for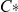 with an increment in input signal. The ability to generate such diverse outputs to a common input, through elementary changes in motif architecture, probably represents a critical feature that confers plasticity during signal processing.

### Motifs can be ranked on the basis of their vulnerability to systemic noise

**Figure 2:**
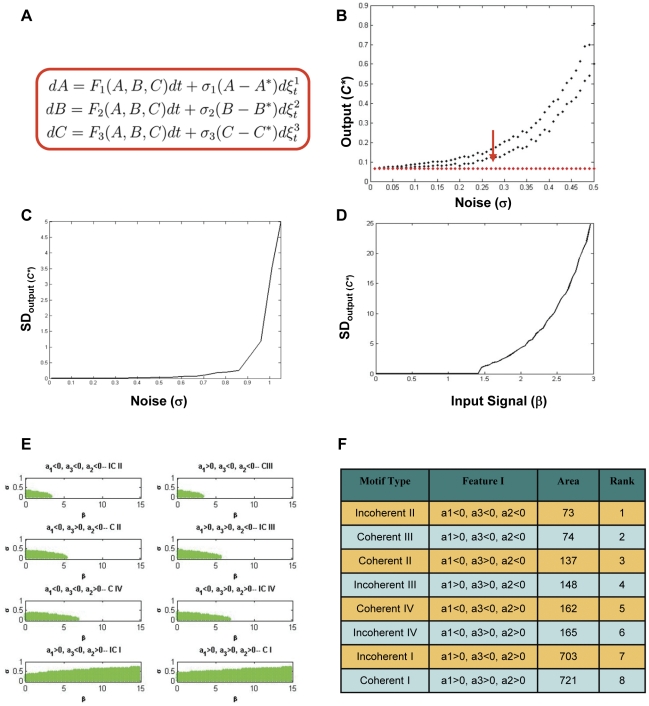
Signal and noise are differentially processed by different FFLs. The model for inclusion of systemic perturbation is shown in Panel A. Here 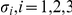 are real constants known as intensities of perturbations. Further, 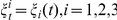 are standard Wiener processes independent of each other. Panel B shows output at node 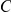
at a given signal as a function of increase in systemic noise (Black dots). Red arrow indicates the threshold noise level at which output becomes divergent (see text). Corresponding output in the absence of noise is also shown (Red dots). As an alternate measure of specific I/O relationship in the presence of systemic noise, standard deviation for the 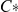 is plotted as a function of 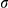 (panel C) or 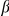 (panel D). Data for plots used in Panel B, C and D were taken for an incoherent type II FFL, for the sake of clarity. Panel E depicts two dimensional (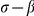) parameter spaces for the stability of the signal output through different FFL architecture. The shaded region shows stability of output at 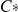. In most cases, an increase in signal lead to higher vulnerability to noise as 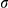
decreases with increase in 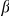 except for the two instances, coherent type I and incoherent type I where the noise threshold increases with increase in signal. Relative ranking of the FFLs on the basis of their vulnerability threshold was calculated by the area of the respective stability zone in 2E and is shown in panel F.

Having established the I/O relationship patterns, we next asked the question of how such motifs are buffered against noise. *In vivo,* cells are continuously exposed to a range of non-specific signals. In addition stochastic processes that introduce mutations or alterations in protein turnover rates also represent non-specific perturbation to the signaling network. To probe this, we incorporated a dispersed stochastic perturbation in our model that could influence any of the components of the motif independent of the signal input (Fig. 2A). The rational for using dispersed perturbation was derived from the fact that a multiplicity of both cell intrinsic and external factors such as cytokines, growth factors, nutrients, environmental stresses, modulations in protein stability, among several others, can potentially influence any of the signaling components through a diverse range of mechanisms [2], [28], [29]. Cumulatively, such perturbations would exert a heterogeneous influence on the basal state of the signaling network. We considered such random influences as systemic perturbations and incorporated these effects into the model as multiplicative Gaussian white noise (Fig. 2A) [5], [10].

Incorporation of systemic perturbations in our model resulted in a marked influence on the nature of the steady state achieved. The output value, instead of a single point observed as observed in the absence of noise, varied widely for several motif types, such that beyond a threshold level of noise the specificity of the response to the input signal was lost. A representative profile of such divergent output response is depicted in Fig. 2B, where 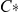 is calculated for an incoherent type II FFL. Beyond a threshold, multiple values distributed over a wide range were obtained. That is, the correlation between input and output was lost and the output became entirely unpredictable (Fig. 2B). The threshold values were calculated analytically (Theorem 1), and it was defined by the point where divergent steady state output responses were initiated (Fig. 2B). Briefly, Theorem 1 defines the relationship between the noise intensities with the system parameters in terms of stochastic stability using methods from Ito' differential calculus (Ref, Mao). Given the variations in the 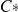 values in the perturbed condition, a precise I/O relationship could not be determined. Instead, a range of values within which the output could lie was obtained. Therefore, we determined the standard deviation for this range as an estimate of increased imprecision in the signal output. At increased perturbation levels, any input-dependent change in output was completely masked by the several-fold higher increase in the standard deviation of the range of these possible values (Fig. 2C). Similar phenomenon was observed when at a constant noise level, input signal was changed (Fig. 2D). The combination of noise and input level could together then describe the vulnerability threshold, beyond which output becomes unstable.

We next examined whether the eight variant architectures for FFLs display distinctions in vulnerability thresholds to such stochastic perturbations. The aim here was to determine whether the fine distinctions in topology between these motifs play any differential role in buffering against noise. Since the vulnerability threshold defines stability of the output we examined the allowed regions of stability for 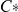, in the two-dimensional parameter space defined by the intensities of the perturbations and the input signal (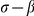, Fig. 2E). A clear distinction in the allowed regions across the individual motifs could be observed with coherent type II and incoherent type II FFLs being revealed as the most resilient by virtue of exhibiting the larger boundary of allowed parameter space in comparison with that of the others (Fig. 2E). On a more quantitative note we estimated the area of the allowed zone of stability in the (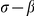) parameter space, and used the obtained values to rank the eight FFL motifs in terms of their vulnerability to stochastic perturbations (Fig. 2F). The results revealed the ranking, in terms of the vulnerability threshold, that described the incoherent type II FFL as the most sensitive and the coherent type I as the least vulnerable to systemic perturbations (Fig. 2F). The remaining motifs were graded between these two extremes. An important aspect here was to ascertain the robustness of the relative ranking of various FFLs to changes in parameter values. Also we wanted to ensure that the rankings hold good under different kinetic laws governing the regulatory behavior. We therefore began with changing the parameter values (a_1_, a_2_ and a_3_), and calculated rank of the eight FFLs in terms of the area under (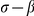) plot. The analysis provided some intriguing observations. Four out of eight motifs (CI, CIII, ICI, and ICII) retained their ranking across the entire range of magnitude for a_1_, a_2_ and a_3_ while other four (ICIV, CII, CIV and ICIII) swapped rankings.

We also performed these analyses using Michaelis-Menten kinetic law and established that relative stability thresholds for various motifs were independent of the magnitude of parameters a_1_, a_2_ and a_3_ under both mass action and Michaelis-Menten kinetics (Figure S1). While, at one level, these findings confirm distinctions in functional properties to individual motifs, they further extend by highlighting that the architecture of a motif also plays a critical role in defining its sensitivity to random perturbations. Such an interpretation then automatically implies that the manner in which various motifs are organized in a signaling network would impact on the overall vulnerability of the network against non-specific perturbations.

### Higher order organization of Motifs exhibits specific design principle

**Figure 3:**
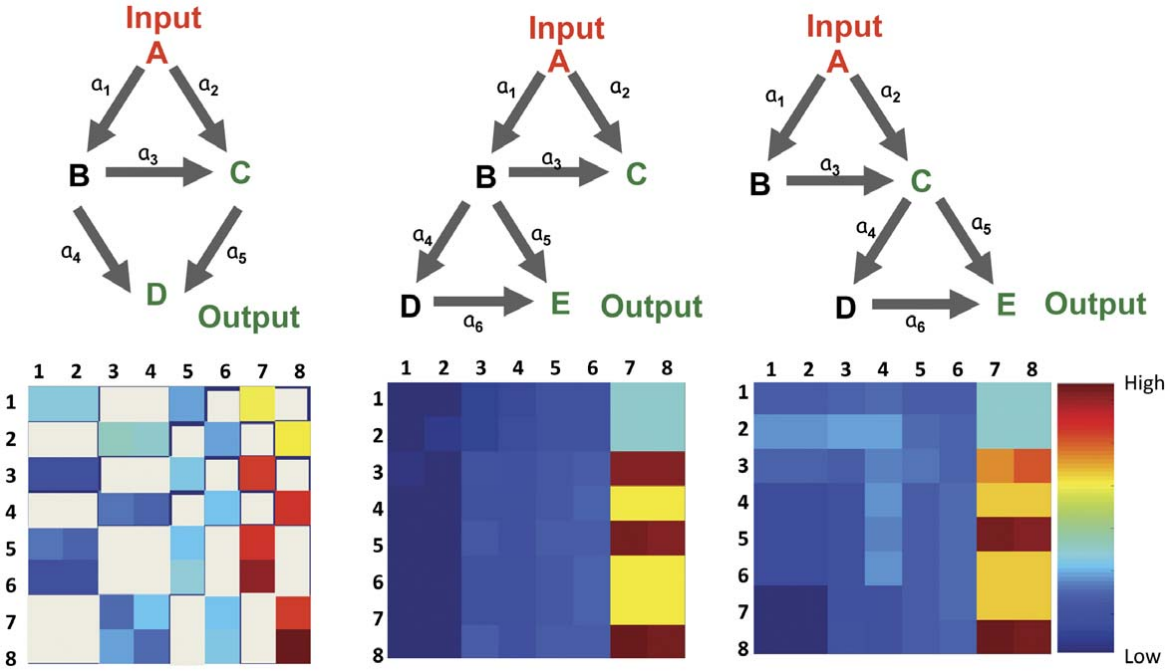
Meta-organization of motifs defines vulnerability and robustness hot-spots in the network. Three possible ways of alternate arrangement of 3 node FFLs are shown in the upper section of Panel A. Corresponding analysis of all possible combinations revealed that diverse robustness patterns could be obtained as a result of permutative combination of these motifs (lower section, Panel 3A). For each combination here, area was calculated in the (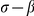) parameter space and was used to plot the pseudo-color maps shown. The color bar at the extreme right shows relative high and low values. Note that the motif organization at the extreme left has only five links where signs could be changed, giving rise to 2^5^ i.e. 32 combinations. Combinations not possible in this organization are shown as gray. The other two organizations could give 64 different combinations. A table ranking various combinations of alternately placed FFLs is presented in Table S1.

Accordingly then, we next asked whether any specific pattern of organization could be detected, for the motifs within a network. To address this at the first level, we continued to restrict our focus on the 3-node FFLs and examined for any preferred order of their organization, in instances where two FFLs occur in tandem. The three different arrangements that are potentially possible in such cases are shown in Figure 3. In spite of the limited set of combination tested here the diversity in robustness threshold generated was remarkable (Fig. 3, lower panel). Therefore clearly in a large signaling network, how different kinds of motifs are organized would determine both the overall robustness and output specificity of the network. Specifically taking cues from Figure 3, formation of high threshold motifs at the effectors end of the network would ensure response specificity as well as diversity. At the same time simply by altering the order in which motifs are organized downstream to input, different I/O relationship could also be achieved. One possible way of altering the order of motif organization could be differential recruitment of various signaling molecule downstream to the receptor.

**Figure 4:**
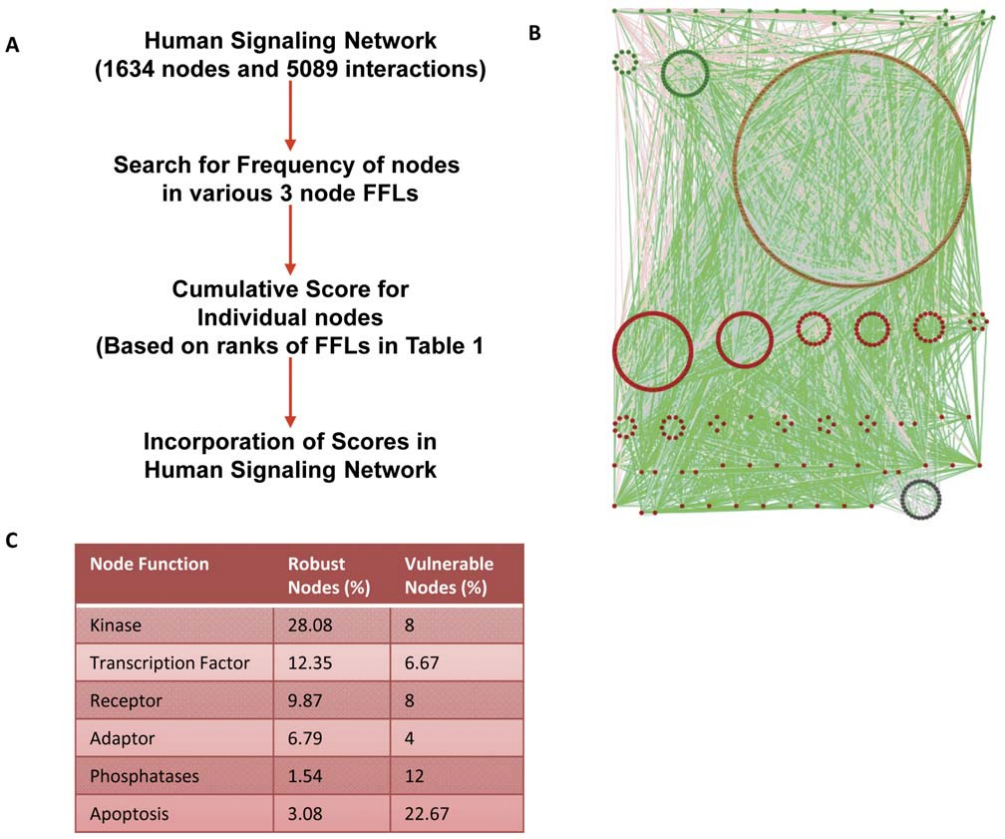
Scoring of signaling intermediates for their vulnerability to noise in a human signaling network. Using the ranking of FFLs shown in Figure 2E, a scoring strategy was developed for the participant nodes (Panel 4A). Then in a real human cancer signaling network, this strategy was applied to score each of nodes in the network. The cancer signaling network sorted on the basis of aggregate score of component nodes is represented in Panel B. distribution of vulnerable (blue), robust (red) and intermediate nodes (yellow and green) are shown in the network. Links in pink are activating interactions while those in blue are inhibitory. Neutral associations are displayed in grey. Classification of robust and vulnerable nodes into various functional categories is shown in Panel C and further listed in Table S3.

To verify these findings, we selected a manually curated, published human signaling network consisting of 1634 nodes and 4888 regulatory interactions [30]. Since nodes with higher connectivity are important for regulating plasticity [17], we wanted to identify whether any specific pattern exists in distribution of signaling molecules across various motifs. We developed an algorithm in MTLAB to measure the frequency of occurrence of a given node in the eight possible three-node FFLs. This was then integrated with the vulnerability ranking of the individual FFLs as outlined in Figure 4A (see ‘Methods’). The resulting scores obtained helped to identify nodes that were overrepresented in either the vulnerable or the robust FFLs (Table S2), and such nodes were described as vulnerability hot spots (VHS) or robustness hot spots (RHS) respectively. Subsequent to this exercise, we reasoned that the distribution pattern of VHS versus RHS in the signaling network would provide insights into its regulatory features and, in particular, the question of how the antithetical properties of robustness and sensitivity are integrated. An analysis of the human signaling network yielded an aggregate of 324 RHS and 75 VHS and this is represented in Figure 4B. While this bias towards RHS - relative to VHS - was not surprising considering robustness displayed by biological signaling networks, a further analysis of these nodes revealed additional features of interest. The RHS were primarily composed of receptors and receptor proximal kinases (Fig. 4C). In contrast, molecules directly regulating cellular responses like apoptosis (Bad, Bcl2, Bax, and other Bcl family members) and phosphatases (MKP1, MKP2, MKP3, MKP5, PPP1CC, PPP2R5C etc) terminal cell-cycle regulatory molecule (CDK4) and ubiquitin conjugating enzymes dominated the constitution of VHS (Fig. 4C).

**Figure 5:**
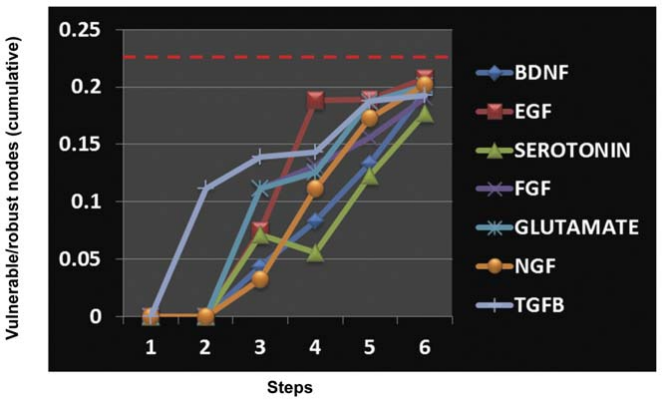
A novel design principle for signaling network organization. Downstream to seven different ligands, we counted number of robust and vulnerable nodes at every step. Ratio of cumulative numbers of vulnerable to robust nodes at every step downstream to any receptor was calculated and is shown in the figure. Enrichment of receptor proximal steps with robust nodes is evident here. The red dotted line represents overall network ratio of robust to vulnerable nodes. This shows that contrary to the network average, early steps of the network are significantly enriched with the robust nodes.

These findings were particularly intriguing in that they suggested the existence of a possible organizational principle for motifs in signaling networks. That is, the enrichment of RHS at the receptor and its proximal levels would confer heightened robustness for filtering out signal from noise As opposed to this, enrichment of VHS within the effector components that define signal output would ensure that the resulting cellular responses are both buffered against noise as well as display sufficient flexibility to ensure divergent cellular responses. This functionally segregated polarization of VHS and RHS may then potentially explain incorporation of the opposing properties of sensitivity and robustness by signaling networks. That the observed segregation of VHS and RHS in the human cancer-signaling network may in fact represent a more general principle was supported by our subsequent analysis of signaling networks downstream of receptors to seven different ligands. These were EGF, TGFB, IL1B, NGF, FGF, BDNF and Glutamate. In all of these cases, we traced the paths downstream of the corresponding receptor. While tracing the paths downstream to these ligands, we ensured directions of the interaction were also accounted for by using our own algorithm developed in MATLAB (see ‘Methods’ for detail). An empirical examination of these networks clearly revealed a biased enrichment of RHS among the receptor proximal components. This observation could be further confirmed by calculating the ratio of robust to vulnerable nodes at each step in the pathway (Fig. 5). A significantly higher presence of RHS in the receptor-proximal steps was clearly detected in all the eight instances studied (Fig. 5).

## Discussion

Biological systems display remarkable robustness despite being surrounded with an environment consisting of diverse physical and physiological stimuli. Some of the attributes that impart robustness to both external and internal perturbations include topological features of the signaling network [31]. The topological features can further be weighted in their magnitude of influence depending on net concentration of the constituent nodes as well as stochastic variations in their level owing to various intrinsic mechanisms [32]. How these features contribute towards overall robustness and sensitivity of biological networks are however not well understood. In the present study, using a very simple three-node feed-forward loops we have shown that depending on the nature of interaction among the constituent nodes in a topological framework, a system might vary between being highly vulnerable or extremely robust for a given input in the presence of inherent stochasticity at each of the nodes. One of the most key advancement in our understanding through this study is the revelation that the topological features like feed-forward loops could be ranked in terms of their role for a common biological property. Next we show that in a signaling network, if we estimate frequency of occurrence of any given node to different kinds of FFLs, they can be scored for their robustness quotient. Interestingly, we observed a level of segregation in the distribution of robust and vulnerable function to molecules known for their different biological functions. On a simpler note, the present study reveal a simple yet elegant design principle that could potentially explain many of the complex traits exhibited by signaling networks. The most significant of these is that the selective positioning of RHS in the receptor-proximal and that of VHS in the effector component-proximal segments facilitates the balance between robustness and diversity of the overall system. It is also likely that the concentration of RHS in the neighborhood of the receptor represents an evolutionary principle that allows cells to evolve more complex networks further downstream, in response to the increasing repertoire of extracellular cues. It was further supported by a detailed look at the composition of molecules at each of the steps. Thus, initial steps downstream to various receptors were enriched with kinases while later steps gradually got enriched with TF regulators and apoptosis regulators. From the contrary view though, the localization of RHS would also suggest that aberrations in functioning of the signaling network leading to disease would largely derive from mutations in the RHS sub-set of signaling nodes. Such an interpretation is indeed supported by the fact that most genes whose mutations are associated with cancer (e.g. ABL1, BRCA1, SRC, ATM, BRAF, PTEN, TGFBR2, EGFR, RB, p53, SMAD4 etc) in fact belong to the category of RHS described here.

We recognize that while our present study was restricted to three-node FFLs, other categories of motifs are also present in the signaling network. Some of these are Feed Back Loops, Four-node FFLs, and Bifans among others. Consequently, an extension of the analysis described here to incorporate all such motifs can clearly be expected to provide many additional insights into the regulatory aspects of signal transduction. From the standpoint of the present study, however, we believe that its highlight is the revelation that a defined principle exists for the higher-order organization of FFLs and, perhaps, motifs in general. Further, our demonstration that this organization impacts on the thresholds governing sensitivity to stimuli and robustness to perturbations is also of particular significance. These findings, therefore, have important implications for understanding both the evolutionary aspects of network design, as well as the etiology of several diseases.

## Methods

### Deterministic model

The model represents a three node signaling motif, Node 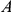 receives the input signal which then influences the output node 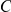 either directly or via additional regulatory node 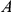. Parameters governing the relationship between the nodes are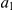, 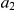
and 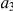 (Fig. 1A), and by simply changing their signs, eight different three-node FFL motif architectures could be described. Individual nodes were modeled for their activated form by defining their basal activation value as 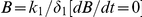 and 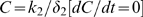.
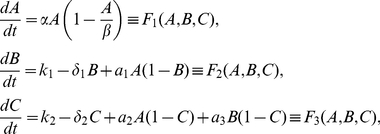
(1)


With initial conditions
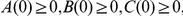


### Steady state analysis

The system (1) has a positive steady state 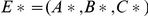 given by
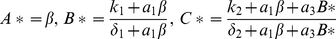



Jacobian matrix of the system (1) around the interior equilibrium point 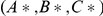 is given by
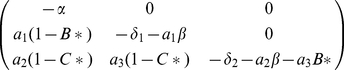
(2)


Since the eigenvalues associated with the matrix (2) are negative real numbers, so the interior equilibrium point 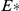 is always stable.

### Stochastic model



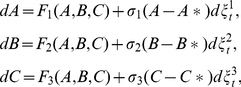
(3)Where, 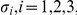 are real constants and known as the intensities of fluctuations, 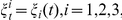 are standard Wiener processes, independent of each other. We consider (3) as an Ito stochastic differential system of type
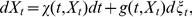
(4)

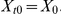



In the equation
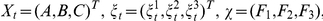


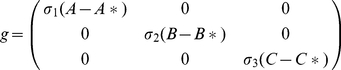
(5)


The linearized version of (4) at 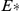 is given by
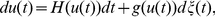
(6)


Where,
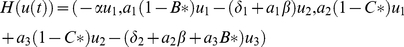
(7)

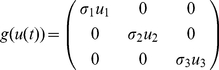
(8)


### Theorem

Assuming that det(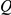) *>0*, where 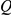 is given in (9) it is observed that the zero solution of system (5) is asymptotically mean square stable if
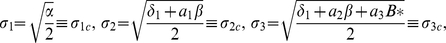
and
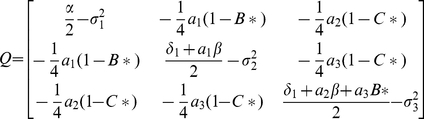
(9)


### Scoring for vulnerability of nodes

We devised an indigenous method to estimate the cumulative vulnerability score for each of the nodes in a signaling network. Suppose *N* denotes the node number and *K_i_* denotes the number of times the *N* ^th^ node occurs in the *i* ^th^ rank. Let *S_i_* be the score given to the *i* ^th^ rank such that 
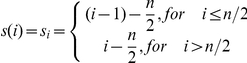
 where *n* denotes the total number of motif types (eight in the present study).

Then the cumulative score given for *N* ^th^ node is
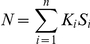



Nodes with the cumulative score greater than *n/2* was then identified as robust nodes while those with less than *–n/2* were classified as vulnerable nodes.

### Algorithm for finding downstream paths of the corresponding receptors

A square matrix *A(i,j)* is made from the network where 
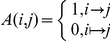


The size of the matrix is the total number of interactions in the network. Then Starting with a node (ligand under investigation), say number *n*, we search for the *n* ^th^ row. We collect all the columns *j’s* such that *A(n,j) = 1*, in a matrix *A1*. This *A1* matrix denotes the nodes coming in the second step in the downstream path of the ligand *n.* With each *j*’s collected in matrix *A1* are now treated as the starting node and for each of them we follow the last step to get new set of nodes. The resulting nodes are collected in another matrix *A2*, which denotes the nodes coming in the third step in the downstream path of the ligand *n.* This process is repeated until we get matrix *A6* with nodes coming in the sixth step in the downstream path of the ligand *n.*

## Supporting Information

Figure S1
**Ranking of motifs is independent of the kinetic law used.** In the study, motifs were ranked depending on the stability area in the (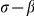) parameter space. Since the original model considered simplistic mass action kinetics, we also performed similar analysis using the Michaelis-Menten kinetic law. A comparison of the relative ranking of the eight motifs under two different governing kinetic laws is shown in Figure S1A (top panel). Lower panel describes the model under the two different kinetic laws used. Figure S1B describes that the relative ranking of five out of the eight motifs are independent of the magnitude of parameters a_1_, a_2_ and a_3_ under both the kinetic law conditions (see text for details). Note that the entire analyses using the two kinetic laws were performed with the same set of parameter values.(TIF)

Table S1 **Meta-organization of FFLs and resulting vulnerability thresholds.** Table ranks all the possible organizations across the three combinations (see Fig. 3) in terms of their vulnerability threshold. A higher threshold rank would therefore mean a more robust organization.
(PDF)

Table S2 **Scoring of nodes for their vulnerability based on the frequency of occurrences in various FFLs.** For each of the 1604 nodes (Rows) in the human cancer signaling network, frequency of their occurrence in various FFLs were calculated. Motifs are shown here as Rank 1 to Rank 8 (Fig. 2E).
(PDF)

Table S3 **Functions assigned to the genes in the signaling network.** Function of all the nodes present in the human signaling network is listed in this table. This data was directly used from an already published manuscript (30).
(PDF)
