# Supplementary figures and images for: Unraveling the Design Principle for Motif Organization in Signaling Networks
Source: PLoS One. 2011 Dec 2;6(12):e28606. doi: 10.1371/journal.pone.0028606 (PMC3228783; doi:10.1371/journal.pone.0028606)

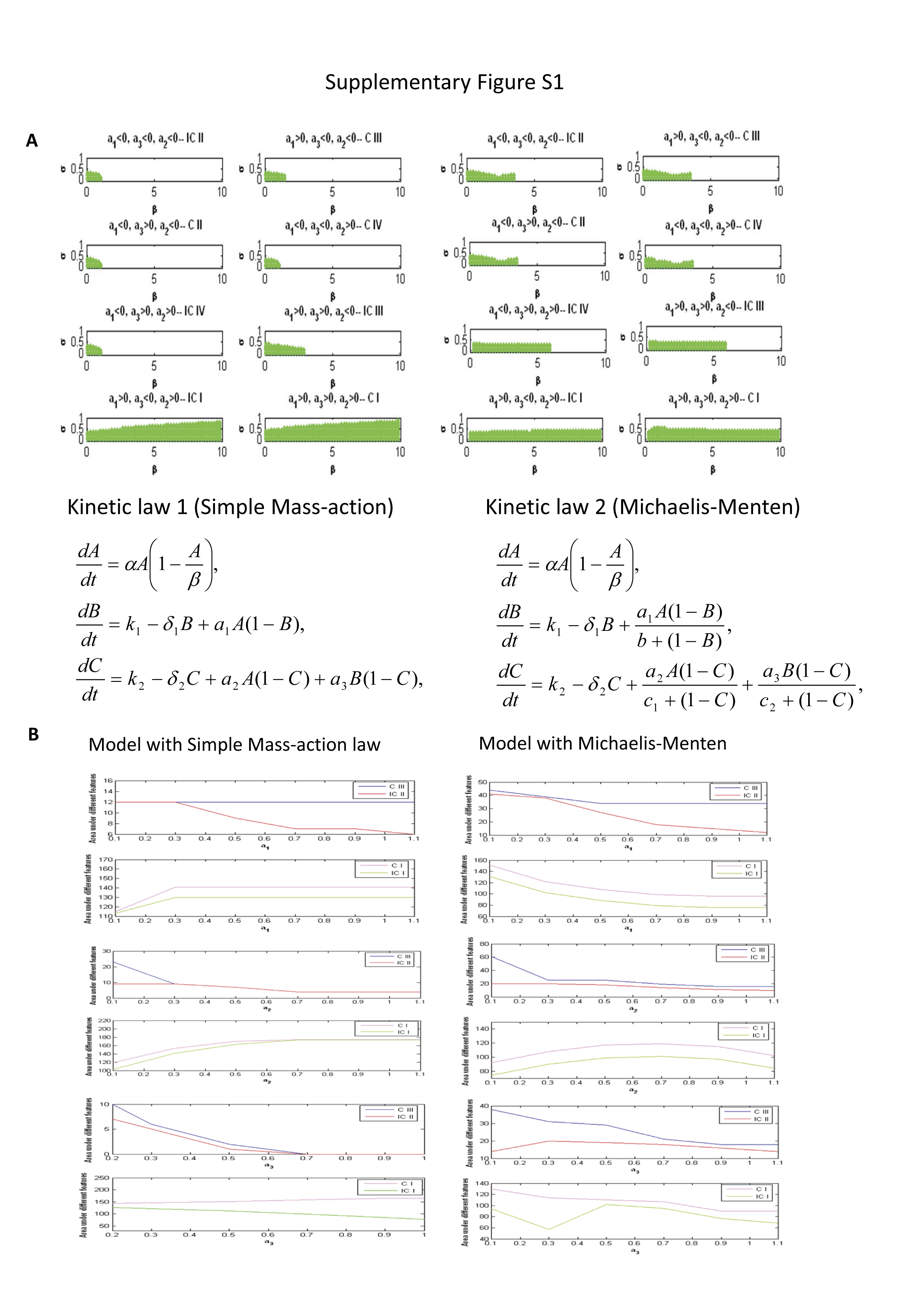

Supplement: Figure S1 — Ranking of motifs is independent of the kinetic law used. In the study, motifs were ranked depending on the stability area in the () parameter space. Since the original model considered simplistic mass action kinetics, we also performed similar analysis using the Michaelis-Menten kinetic law. A comparison of the relative ranking of the eight motifs under two different governing kinetic laws is shown in Figure S1A (top panel). Lower panel describes the model under the two different kinetic laws used. Figure S1B describes that the relative ranking of five out of the eight motifs are independent of the magnitude of parameters a1, a2 and a3 under both the kinetic law conditions (see text for details). Note that the entire analyses using the two kinetic laws were performed with the same set of parameter values. (TIF) [file pone.0028606.s001.tif]
