# Supplementary material for: Unraveling the Design Principle for Motif Organization in Signaling Networks
Source: PLoS One. 2011 Dec 2;6(12):e28606. doi: 10.1371/journal.pone.0028606 (PMC3228783; doi:10.1371/journal.pone.0028606)
Supplement: Table S1 — Meta-organization of FFLs and resulting vulnerability thresholds. Table ranks all the possible organizations across the three combinations (see Fig. 3) in terms of their vulnerability threshold. A higher threshold rank would therefore mean a more robust organization. (PDF) [file pone.0028606.s002.pdf]

Supplementary Table S1

A.

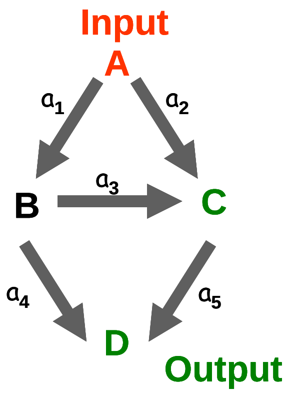

	First FFL	a1<0, a3<0, a2<0	a1>0, a3<0, a2<0	a1<0, a3>0, a2<0	a1>0, a3>0, a2<0	a1<0, a3<0, a2>0	a1<0, a3>0, a2>0	a1>0, a3<0, a2>0	a1>0, a3>0, a2>0
Second FFL		Incoherent II	Coherent III	Coherent II	Incoherent III	Coherent IV	Incoherent IV	Incoherent I	Coherent I
a3<0, a5<0, a4<0	Incoherent II	129	130	0	0	107	0	223	0
a3>0, a5<0, a4<0	Coherent III	0	0	143	140	0	107	0	232
a3<0, a5>0, a4<0	Coherent II	42	40	0	0	125	0	296	0
a3>0, a5>0, a4<0	Incoherent III	0	0	90	79	0	119	0	305
a3<0, a5<0, a4>0	Coherent IV	90	79	0	0	119	0	305	0
a3<0, a5>0, a4>0	Incoherent IV	44	40	0	0	131	0	344	0
a3>0, a5<0, a4>0	Incoherent I	0	0	84	118	0	119	0	299
a3>0, a5>0, a4>0	Coherent I	0	0	103	83	0	120	0	364

B.

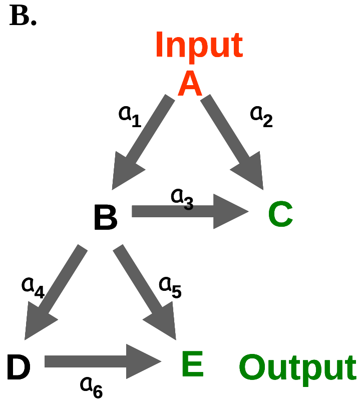

	First FFL	a1<0, a3<0, a2<0	a1>0, a3<0, a2<0	a1<0, a3>0, a2<0	a1>0, a3>0, a2<0	a1<0, a3<0, a2>0	a1<0, a3>0, a2>0	a1>0, a3<0, a2>0	a1>0, a3>0, a2>0
Second FFL		Incoherent II	Coherent III	Coherent II	Incoherent III	Coherent IV	Incoherent IV	Incoherent I	Coherent I
a4<0, a6<0, a5<0	Incoherent II	56	59	84	95	130	130	240	240
a4>0, a6<0, a5<0	Coherent III	53	74	84	104	129	129	240	240
a4<0, a6>0, a5<0	Coherent II	60	53	128	117	143	148	536	531
a4>0, a6>0, a5<0	Incoherent III	52	53	108	111	145	160	372	372
a4<0, a6<0, a5>0	Coherent IV	52	53	142	117	147	150	538	534
a4<0, a6>0, a5>0	Incoherent IV	52	53	108	111	145	160	372	372
a4>0, a6<0, a5>0	Incoherent I	52	53	108	111	145	160	372	372
a4>0, a6>0, a5>0	Coherent I	52	53	147	120	152	152	552	537

C.

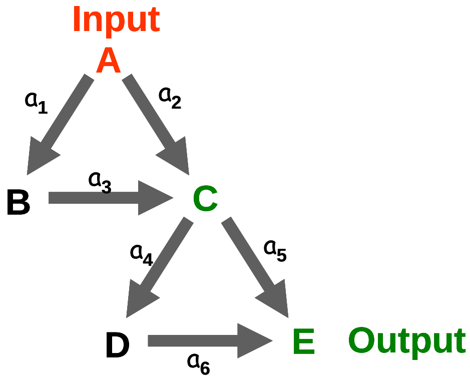

	First FFL	a1<0, a3<0, a2<0	a1>0, a3<0, a2<0	a1<0, a3>0, a2<0	a1>0, a3>0, a2<0	a1<0, a3<0, a2>0	a1<0, a3>0, a2>0	a1>0, a3<0, a2>0	a1>0, a3>0, a2>0
Second FFL		Incoherent II	Coherent III	Coherent II	Incoherent III	Coherent IV	Incoherent IV	Incoherent I	Coherent I
a4<0, a6<0, a5<0	Incoherent II	144	143	156	159	145	145	240	240
a4>0, a6<0, a5<0	Coherent III	183	183	192	192	162	152	240	240
a4<0, a6>0, a5<0	Coherent II	154	154	149	173	171	152	402	438
a4>0, a6>0, a5<0	Incoherent III	97	97	107	181	145	161	371	372
a4<0, a6<0, a5>0	Coherent IV	97	97	107	177	144	158	527	520
a4<0, a6>0, a5>0	Incoherent IV	97	97	107	181	144	161	371	372
a4>0, a6<0, a5>0	Incoherent I	51	53	117	111	146	161	372	372
a4>0, a6>0, a5>0	Coherent I	51	53	121	110	148	150	537	527
