# Supplementary material for: Unraveling the Design Principle for Motif Organization in Signaling Networks
Source: PLoS One. 2011 Dec 2;6(12):e28606. doi: 10.1371/journal.pone.0028606 (PMC3228783; doi:10.1371/journal.pone.0028606)
Supplement: Table S2 — Scoring of nodes for their vulnerability based on the frequency of occurrences in various FFLs. For each of the 1604 nodes (Rows) in the human cancer signaling network, frequency of their occurrence in various FFLs were calculated. Motifs are shown here as Rank 1 to Rank 8 (Fig. 2E). (PDF) [file pone.0028606.s003.pdf]

Supplementary Table S2

Node	Rank 1	Rank 2	Rank 3	Rank 4	Rank 5	Rank 6	Rank 7	Rank 8	Score
1	1	1	2	2	2	1	1	1	0
2	0	0	0	0	0	0	0	0	0
3	0	0	0	0	0	0	0	0	0
4	0	0	0	0	0	0	0	0	0
5	0	0	0	0	0	0	0	0	0
6	0	0	0	0	0	0	0	1	1
7	0	0	0	0	0	0	0	0	0
8	0	0	0	0	0	0	0	1	1
9	0	0	1	0	0	1	2	2	4
10	0	0	0	0	0	0	0	0	0
11	0	0	0	0	0	0	0	0	0
12	0	0	0	0	0	0	0	0	0
13	0	0	0	0	0	0	0	0	0
14	0	0	0	0	0	0	0	0	0
15	0	0	0	0	0	0	0	0	0
16	0	0	0	0	0	0	0	0	0
17	0	0	0	0	0	0	0	0	0
18	0	0	0	0	0	0	0	0	0
19	0	0	0	0	0	0	0	0	0
20	0	0	0	0	0	0	0	1	1
21	0	0	0	0	0	0	0	1	1
22	2	3	7	4	2	10	3	22	20
23	0	0	0	0	0	0	0	0	0
24	0	0	0	0	0	0	0	0	0
25	0	0	0	0	0	0	0	0	0
26	0	0	0	0	0	0	0	0	0
27	0	0	0	0	0	0	0	0	0
28	1	1	1	1	0	0	2	4	4
29	0	0	1	0	0	0	0	0	0
30	0	0	0	0	0	0	0	0	0
31	0	0	0	0	0	0	0	0	0
32	0	0	0	0	0	0	0	0	0
33	0	0	0	0	0	0	0	0	0
34	0	0	0	0	0	0	0	0	0
35	0	0	0	0	0	0	0	0	0
36	0	0	0	0	0	0	0	2	2
37	0	0	0	0	0	0	0	0	0
38	0	0	0	0	0	0	0	0	0
39	0	0	0	0	0	0	0	0	0
40	0	0	0	0	0	0	0	2	2
41	0	0	0	0	0	0	0	0	0
42	0	0	0	0	0	0	0	0	0
43	0	0	0	0	0	0	0	0	0
44	0	0	0	0	0	0	0	0	0
45	0	0	0	0	0	0	0	0	0
46	0	0	0	0	0	0	0	5	5
47	0	0	0	0	0	0	0	8	8
48	0	0	0	0	0	2	1	8	9
49	0	0	0	0	0	0	1	1	2
50	0	0	0	0	0	0	1	11	12
51	0	0	0	0	0	0	0	0	0

52	0	0	0	0	0	0	0	0	0
53	0	0	3	0	0	0	1	2	3
54	0	0	0	0	0	0	0	0	0
55	18	11	17	6	14	9	8	6	-15
56	4	21	3	4	15	5	4	11	-10
57	10	24	11	4	24	11	6	17	-11
58	30	32	31	4	32	11	11	2	-49
59	28	33	17	4	23	6	5	1	-55
60	1	9	1	3	6	0	1	10	1
61	3	3	3	1	3	0	1	0	-5
62	0	0	2	2	1	1	0	2	2
63	0	0	0	0	0	0	0	2	2
64	0	0	0	0	0	1	0	0	0
65	0	0	0	0	0	0	0	0	0
66	0	0	0	0	0	0	0	3	3
67	0	0	0	0	0	0	0	0	0
68	0	0	0	0	0	0	0	1	1
69	0	0	0	0	0	0	0	0	0
70	0	0	0	0	0	0	0	2	2
71	0	1	0	2	0	1	1	18	18
72	0	0	0	0	0	1	0	1	1
73	0	0	1	2	1	2	2	3	5
74	0	0	0	0	0	1	1	4	5
75	0	1	0	0	0	0	0	6	5
76	0	0	0	0	0	0	0	2	2
77	0	1	0	0	0	0	0	0	-1
78	0	0	0	0	0	1	0	3	3
79	0	1	0	0	0	0	0	0	-1
80	0	0	0	0	0	0	0	0	0
81	0	0	0	0	0	0	1	1	2
82	0	0	0	0	0	0	0	0	0
83	0	0	0	0	0	0	0	2	2
84	0	0	0	0	0	0	0	0	0
85	0	0	0	2	0	0	2	1	3
86	0	0	0	0	0	0	0	0	0
87	7	16	14	5	14	11	6	13	-4
88	0	2	0	0	1	3	1	1	0
89	0	11	4	2	0	4	1	3	-7
90	0	5	2	2	0	1	1	4	0
91	6	12	6	1	0	6	0	2	-16
92	0	0	0	0	0	0	0	0	0
93	0	2	0	0	0	0	2	3	3
94	0	0	0	0	0	1	2	9	11
95	0	0	0	0	0	0	0	0	0
96	1	2	0	0	0	1	0	1	-2
97	0	0	0	2	0	1	0	0	0
98	0	0	1	0	1	1	1	0	1
99	0	0	0	0	0	0	0	0	0
100	0	0	0	0	0	0	0	0	0
101	0	0	0	0	0	0	0	2	2
102	0	0	0	0	0	0	0	0	0
103	0	0	0	0	0	0	0	0	0
104	0	0	0	0	0	0	0	0	0

105	0	0	0	0	0	0	0	0	0
106	0	0	0	0	0	0	0	0	0
107	0	0	0	0	0	0	0	0	0
108	0	0	0	0	0	0	0	0	0
109	0	0	0	0	0	0	0	0	0
110	0	0	0	0	0	0	0	0	0
111	0	0	0	0	0	0	0	0	0
112	0	0	0	0	0	0	0	0	0
113	0	0	0	0	0	0	0	0	0
114	0	0	0	0	0	0	0	0	0
115	0	0	0	0	0	0	0	0	0
116	0	0	0	0	0	0	0	3	3
117	0	0	0	0	0	0	0	0	0
118	0	1	0	1	0	1	0	0	-1
119	0	1	1	0	0	1	2	2	3
120	1	1	0	0	0	0	0	0	-2
121	0	0	0	1	0	0	0	0	0
122	0	0	0	0	0	0	0	0	0
123	0	0	0	0	0	0	0	0	0
124	0	0	0	0	0	0	1	0	1
125	0	0	0	0	0	0	0	0	0
126	0	0	0	0	0	1	0	2	2
127	0	0	0	0	0	1	0	4	4
128	0	0	0	0	0	0	0	0	0
129	0	0	0	0	0	0	0	0	0
130	0	0	0	0	0	0	0	2	2
131	0	0	2	2	1	1	0	1	1
132	0	1	0	0	0	0	0	0	-1
133	0	0	0	3	0	0	1	18	19
134	0	0	0	0	0	0	0	1	1
135	0	0	0	0	0	0	0	0	0
136	0	0	0	0	0	0	0	0	0
137	0	0	0	0	0	0	0	0	0
138	0	0	0	0	0	0	0	0	0
139	0	0	0	0	0	1	0	0	0
140	0	0	0	0	0	0	0	0	0
141	0	0	0	0	0	0	0	0	0
142	0	0	0	1	0	0	0	0	0
143	0	0	0	0	0	0	0	0	0
144	1	6	0	2	1	1	2	16	11
145	1	1	0	0	0	0	2	0	0
146	0	0	0	0	0	0	0	4	4
147	0	0	0	0	0	0	0	0	0
148	1	0	0	0	0	0	0	0	-1
149	0	0	0	0	0	0	0	0	0
150	0	0	0	2	0	1	0	1	1
151	0	0	0	0	0	0	0	0	0
152	0	0	0	0	0	0	0	0	0
153	0	0	0	0	0	0	0	0	0
154	1	1	4	1	0	1	3	2	3
155	0	0	0	0	0	0	0	0	0
156	0	0	0	0	0	0	0	0	0
157	0	0	0	0	0	0	0	0	0

158	0	0	0	0	0	0	0	0	0
159	0	0	0	0	0	0	0	0	0
160	0	0	0	0	0	0	0	0	0
161	0	0	0	0	0	0	0	0	0
162	0	0	0	0	0	0	0	0	0
163	0	0	0	0	0	0	0	0	0
164	0	0	0	3	0	0	0	12	12
165	0	0	0	0	0	0	0	1	1
166	0	0	0	0	0	0	0	1	1
167	0	0	0	0	0	0	0	1	1
168	0	0	0	0	0	0	0	3	3
169	0	0	0	0	0	0	0	0	0
170	0	0	0	0	0	0	0	0	0
171	0	1	0	0	0	0	0	0	-1
172	0	0	0	0	0	0	0	0	0
173	0	0	0	0	0	0	0	0	0
174	0	0	0	0	0	0	0	0	0
175	0	0	0	2	0	0	0	3	3
176	0	0	0	0	0	0	0	0	0
177	0	0	0	0	0	0	0	0	0
178	0	0	0	0	0	0	0	0	0
179	0	0	0	0	0	0	0	0	0
180	1	1	0	0	0	0	0	0	-2
181	0	2	0	1	0	0	0	4	2
182	0	0	0	0	0	0	0	0	0
183	0	0	0	0	0	0	0	0	0
184	0	0	0	0	0	0	0	0	0
185	0	0	0	0	0	0	0	0	0
186	0	0	0	0	0	0	0	0	0
187	0	0	2	3	2	3	3	3	6
188	0	0	0	0	0	0	0	0	0
189	0	0	0	0	0	0	0	0	0
190	0	0	0	1	0	0	0	0	0
191	0	0	0	0	0	0	0	0	0
192	0	0	0	0	0	0	0	0	0
193	0	0	0	0	0	0	0	2	2
194	0	0	0	0	0	0	0	0	0
195	0	0	0	0	0	0	0	0	0
196	0	0	0	0	0	0	0	0	0
197	0	4	0	2	0	1	3	51	50
198	0	0	0	0	0	0	0	0	0
199	0	0	0	0	0	0	0	0	0
200	0	0	0	0	0	0	0	0	0
201	0	0	0	0	0	0	0	0	0
202	0	0	0	0	0	0	0	0	0
203	0	0	0	0	0	0	0	0	0
204	0	0	0	0	0	0	0	0	0
205	0	0	0	0	0	0	0	0	0
206	0	0	0	0	0	0	0	1	1
207	0	0	0	0	0	0	0	1	1
208	0	0	0	0	0	0	0	0	0
209	0	0	0	0	0	0	0	0	0
210	0	0	0	0	0	0	0	0	0

211	0	0	0	2	0	0	0	13	13
212	0	0	0	0	0	0	0	0	0
213	0	0	0	0	0	0	0	0	0
214	0	0	0	0	0	0	0	0	0
215	0	0	0	0	0	0	0	0	0
216	3	0	0	0	0	0	0	4	1
217	0	0	0	0	0	0	0	12	12
218	0	0	0	0	0	0	0	0	0
219	0	5	0	1	0	4	1	34	30
220	2	4	0	5	0	1	2	40	36
221	0	0	0	0	0	0	0	1	1
222	0	0	0	0	0	0	0	0	0
223	0	0	0	0	0	0	0	0	0
224	0	0	0	0	0	0	0	0	0
225	0	0	0	0	0	0	0	0	0
226	0	0	0	0	0	0	0	0	0
227	0	0	0	0	0	0	0	0	0
228	0	0	0	0	0	0	0	0	0
229	0	0	0	0	0	0	0	0	0
230	0	0	0	0	0	0	0	0	0
231	0	0	0	0	0	0	0	0	0
232	0	1	0	0	0	1	1	3	3
233	0	1	0	1	0	3	0	31	30
234	0	0	0	0	0	0	0	0	0
235	0	0	0	0	0	0	0	0	0
236	0	0	0	0	0	0	0	0	0
237	0	0	0	0	0	0	0	1	1
238	0	0	0	0	0	0	0	0	0
239	0	0	0	0	0	0	0	0	0
240	0	0	0	0	0	0	0	0	0
241	0	0	0	0	0	0	0	0	0
242	0	0	0	0	0	1	0	1	1
243	0	0	0	0	0	1	0	1	1
244	0	0	0	0	0	0	0	0	0
245	0	0	0	0	0	0	0	0	0
246	0	0	0	0	0	0	0	0	0
247	0	0	0	0	0	0	0	0	0
248	0	0	0	0	0	0	0	0	0
249	0	0	0	0	0	0	0	1	1
250	0	0	0	0	0	0	0	0	0
251	0	0	0	0	0	0	0	0	0
252	0	0	0	0	0	0	0	0	0
253	0	0	0	3	0	0	2	12	14
254	0	0	0	0	0	0	0	0	0
255	0	1	0	0	0	0	0	0	-1
256	1	2	0	0	0	1	2	6	5
257	0	0	0	0	0	0	0	0	0
258	0	0	0	0	0	0	0	0	0
259	0	0	0	0	0	0	0	0	0
260	0	1	0	0	0	0	0	5	4
261	0	0	0	0	0	0	0	0	0
262	0	0	0	0	0	0	0	0	0
263	0	0	0	0	0	0	0	0	0

264	0	0	0	1	0	2	2	5	7
265	0	0	0	0	0	0	0	0	0
266	0	0	0	1	1	2	0	1	1
267	0	0	0	0	0	0	0	0	0
268	0	0	1	1	0	1	0	8	8
269	0	0	0	0	0	0	1	1	2
270	0	1	0	0	0	0	0	8	7
271	0	0	0	0	0	0	0	0	0
272	0	0	0	0	0	0	0	0	0
273	0	0	0	0	0	0	0	0	0
274	0	0	0	0	0	0	0	0	0
275	0	0	0	1	0	0	0	3	3
276	0	0	0	0	0	0	0	0	0
277	0	0	0	0	0	0	0	0	0
278	0	0	0	0	0	0	0	0	0
279	0	0	0	0	0	0	0	2	2
280	0	0	0	0	0	0	0	0	0
281	0	0	0	0	0	0	0	0	0
282	0	0	0	1	0	2	2	14	16
283	0	0	0	0	0	0	0	2	2
284	0	0	1	1	1	0	0	1	1
285	0	0	0	0	0	0	0	0	0
286	0	0	0	0	0	0	0	0	0
287	0	0	0	0	0	0	0	0	0
288	0	0	0	0	0	0	0	0	0
289	0	1	0	1	0	0	1	7	7
290	0	0	0	0	0	0	0	0	0
291	0	0	0	0	0	0	0	0	0
292	0	0	0	0	0	0	0	0	0
293	0	0	0	0	0	0	0	0	0
294	0	0	0	0	0	0	0	0	0
295	0	0	0	0	0	0	0	0	0
296	0	0	0	0	0	0	0	5	5
297	0	0	0	0	0	0	0	0	0
298	0	0	0	0	0	0	0	1	1
299	0	0	0	0	0	0	0	0	0
300	0	0	0	0	0	0	0	0	0
301	0	0	0	0	0	0	0	0	0
302	0	5	0	0	1	2	1	41	37
303	0	0	0	0	0	0	0	0	0
304	0	0	0	0	0	0	0	0	0
305	0	0	0	0	0	0	0	1	1
306	0	0	0	0	0	0	0	0	0
307	1	3	5	2	1	2	3	2	1
308	0	3	0	0	0	1	4	3	4
309	0	0	0	0	0	0	0	0	0
310	0	0	0	0	0	0	0	0	0
311	0	0	0	4	0	1	5	20	25
312	0	0	0	0	0	4	1	10	11
313	0	0	0	0	0	0	0	0	0
314	0	0	0	0	0	0	0	0	0
315	0	0	0	0	0	0	0	0	0
316	0	0	0	0	0	0	0	0	0

317	0	0	0	0	0	0	0	0	0
318	0	0	0	1	0	0	0	1	1
319	0	1	0	0	0	0	0	1	0
320	0	0	0	0	0	0	0	0	0
321	0	0	0	0	0	0	0	0	0
322	0	0	0	0	0	0	0	0	0
323	0	0	0	0	0	0	0	0	0
324	0	0	0	0	0	0	0	0	0
325	0	0	0	0	0	0	0	0	0
326	0	0	0	0	0	0	0	0	0
327	0	0	0	0	0	0	0	0	0
328	0	0	0	0	0	0	0	0	0
329	0	0	0	0	0	0	0	0	0
330	0	0	0	0	0	0	0	0	0
331	0	0	0	0	0	0	0	0	0
332	0	0	0	0	0	0	0	0	0
333	0	0	0	0	0	0	0	0	0
334	0	0	0	0	0	0	0	0	0
335	0	0	1	0	0	0	0	1	1
336	0	0	0	0	0	0	0	0	0
337	0	0	0	0	0	0	0	0	0
338	0	0	0	0	0	0	0	0	0
339	0	0	0	0	0	0	0	0	0
340	0	0	0	1	0	0	0	0	0
341	0	0	0	0	0	0	0	0	0
342	0	0	1	2	1	4	2	3	5
343	0	1	0	0	0	1	0	0	-1
344	0	0	0	0	0	0	0	1	1
345	0	0	0	0	0	0	0	4	4
346	0	0	0	0	0	0	0	0	0
347	0	0	0	0	0	0	3	10	13
348	0	2	0	1	0	3	7	13	18
349	0	0	0	0	0	0	0	0	0
350	0	0	0	0	0	0	1	3	4
351	0	0	0	0	0	0	1	5	6
352	0	0	0	0	0	0	1	3	4
353	0	0	0	0	0	0	0	0	0
354	0	0	0	0	0	0	0	0	0
355	0	0	0	0	0	0	0	0	0
356	0	0	0	0	0	0	0	0	0
357	0	0	0	0	0	0	0	0	0
358	0	1	0	0	0	1	1	1	1
359	0	0	0	0	0	0	0	2	2
360	0	1	0	0	0	1	1	2	2
361	0	0	0	0	0	0	0	0	0
362	0	0	0	0	0	0	0	0	0
363	0	0	0	0	0	0	0	0	0
364	0	0	0	0	0	0	0	1	1
365	0	0	0	0	0	0	0	0	0
366	0	0	0	0	0	0	0	0	0
367	0	0	0	0	0	0	0	8	8
368	0	0	0	0	0	1	0	2	2
369	0	0	0	0	0	0	0	2	2

370	0	0	0	1	0	0	2	6	8
371	0	0	0	0	0	0	0	0	0
372	0	0	0	0	0	0	0	0	0
373	0	0	1	0	1	0	0	2	2
374	0	0	0	1	0	2	0	2	2
375	0	0	0	0	0	0	0	0	0
376	0	6	0	1	0	0	0	10	4
377	0	4	0	1	0	0	0	11	7
378	2	5	6	8	5	3	7	31	31
379	0	0	0	0	0	1	0	15	15
380	0	0	0	0	0	0	0	0	0
381	0	0	0	0	0	0	0	0	0
382	0	0	0	0	0	0	0	0	0
383	0	0	0	0	0	0	0	0	0
384	0	0	0	0	0	0	0	0	0
385	0	0	0	0	0	0	0	0	0
386	0	0	0	0	0	0	0	0	0
387	0	0	0	0	0	0	0	0	0
388	0	0	0	0	0	0	0	2	2
389	0	1	0	0	0	0	0	2	1
390	0	0	0	0	0	0	0	0	0
391	0	0	0	0	0	0	0	0	0
392	0	0	0	1	0	0	0	0	0
393	0	0	0	0	0	0	0	0	0
394	0	0	0	0	0	0	0	0	0
395	0	0	0	0	0	0	0	0	0
396	0	0	0	0	0	0	0	0	0
397	0	0	0	0	0	0	0	0	0
398	0	0	0	0	0	0	0	0	0
399	0	0	0	0	0	0	0	0	0
400	0	0	0	0	0	0	0	5	5
401	0	0	0	0	0	0	0	0	0
402	0	0	0	0	0	0	0	0	0
403	0	0	0	0	0	0	1	2	3
404	0	0	0	0	0	0	0	0	0
405	0	0	0	0	0	0	0	0	0
406	0	0	0	0	0	0	0	0	0
407	0	5	0	2	0	1	2	24	21
408	0	0	0	0	0	0	0	2	2
409	0	0	0	0	0	0	1	0	1
410	0	0	0	0	0	0	0	0	0
411	0	0	0	0	0	0	0	0	0
412	0	0	0	0	0	0	0	0	0
413	0	0	1	1	1	0	0	0	0
414	0	0	0	1	0	0	0	1	1
415	0	0	0	0	0	0	0	0	0
416	0	0	0	0	0	0	0	0	0
417	0	2	2	4	3	1	3	61	62
418	0	0	0	0	0	0	0	10	10
419	0	0	0	0	0	0	0	13	13
420	0	0	0	0	0	0	0	2	2
421	0	0	0	1	0	0	1	39	40
422	0	0	0	0	0	0	0	0	0

423	0	0	0	0	0	0	0	0	0
424	0	0	0	0	0	1	0	4	4
425	0	0	0	0	0	0	0	0	0
426	0	0	0	0	0	0	0	0	0
427	0	0	0	0	0	0	0	0	0
428	0	0	0	0	0	0	0	0	0
429	0	0	0	0	0	1	0	5	5
430	1	0	0	0	0	0	0	0	-1
431	0	0	0	1	0	0	0	0	0
432	0	0	0	4	0	1	0	4	4
433	0	0	1	2	0	0	0	0	0
434	0	0	0	0	0	0	0	1	1
435	0	0	0	0	0	0	0	1	1
436	0	0	0	0	0	0	0	0	0
437	0	0	0	0	0	0	0	0	0
438	0	0	0	4	0	0	0	2	2
439	0	0	0	0	0	0	0	8	8
440	0	0	0	1	0	0	0	0	0
441	0	0	0	0	0	0	0	1	1
442	0	0	0	1	0	0	0	2	2
443	0	0	0	2	0	1	0	0	0
444	0	0	0	0	0	0	0	0	0
445	0	0	0	0	0	0	0	0	0
446	0	0	0	0	0	0	0	0	0
447	0	1	1	1	0	1	1	7	7
448	0	2	0	0	0	4	6	23	27
449	0	0	0	0	0	0	0	0	0
450	0	0	0	0	0	0	0	0	0
451	0	0	0	0	0	0	0	0	0
452	0	0	0	0	0	0	0	0	0
453	0	0	0	0	0	0	0	0	0
454	0	0	0	0	0	0	0	4	4
455	0	0	0	0	0	0	0	2	2
456	0	0	0	0	0	0	0	0	0
457	0	0	0	0	1	0	2	2	4
458	0	0	0	0	0	0	0	0	0
459	0	0	0	0	0	0	0	0	0
460	0	0	0	0	0	0	0	0	0
461	0	0	0	0	0	0	0	0	0
462	0	0	0	0	0	0	0	0	0
463	0	0	0	0	0	0	0	9	9
464	0	0	1	0	1	1	0	0	0
465	0	0	0	0	0	0	0	0	0
466	0	0	0	0	0	0	0	1	1
467	0	0	0	0	0	0	0	0	0
468	0	0	0	0	0	1	0	2	2
469	0	0	0	0	0	0	0	0	0
470	0	0	0	0	0	0	0	7	7
471	0	0	1	1	2	1	0	0	0
472	0	0	0	0	0	0	0	0	0
473	0	0	0	0	0	0	0	0	0
474	0	0	0	0	0	0	0	0	0
475	0	0	0	0	0	0	0	0	0

476	1	3	1	0	0	1	0	1	-3
477	0	0	0	0	0	0	0	0	0
478	0	0	0	0	0	0	0	1	1
479	0	0	0	1	0	0	0	0	0
480	0	0	0	0	0	0	0	0	0
481	0	0	0	0	0	0	0	0	0
482	0	2	1	1	0	1	2	35	35
483	0	0	0	0	0	0	0	1	1
484	1	6	11	2	12	5	2	13	8
485	0	0	0	0	0	0	0	0	0
486	0	0	0	0	0	0	0	0	0
487	0	0	0	0	0	0	0	0	0
488	0	0	0	0	0	0	0	5	5
489	0	0	0	0	0	0	0	0	0
490	0	0	0	0	0	0	0	8	8
491	0	0	0	0	0	0	0	0	0
492	0	0	0	0	0	0	0	0	0
493	0	0	2	3	0	2	1	16	17
494	0	0	0	0	0	0	0	1	1
495	0	0	0	0	0	0	0	0	0
496	0	0	0	0	0	0	0	2	2
497	0	0	1	0	0	0	0	0	0
498	0	0	0	0	0	0	0	0	0
499	0	0	0	0	0	0	0	0	0
500	0	1	0	0	0	0	0	0	-1
501	0	0	0	0	0	0	0	0	0
502	0	0	0	0	0	0	0	0	0
503	0	0	0	0	0	0	0	0	0
504	0	0	0	0	0	0	0	0	0
505	0	0	0	0	0	0	0	0	0
506	0	0	0	0	0	0	0	1	1
507	0	0	0	1	0	1	0	4	4
508	0	0	0	0	0	1	0	0	0
509	0	0	0	0	0	0	0	0	0
510	0	0	0	0	0	0	0	0	0
511	0	0	0	0	0	1	0	0	0
512	0	0	0	0	0	0	0	0	0
513	0	0	0	0	0	0	0	0	0
514	0	0	0	0	0	0	0	1	1
515	0	4	2	5	1	5	1	44	41
516	0	0	0	0	0	0	0	0	0
517	0	0	0	0	0	0	0	1	1
518	0	0	0	2	0	0	1	21	22
519	0	0	0	1	0	0	1	6	7
520	0	0	0	0	0	0	0	0	0
521	0	0	0	0	0	0	1	4	5
522	0	0	0	3	0	3	2	31	33
523	0	0	0	0	1	2	2	3	5
524	0	0	0	0	0	0	0	0	0
525	0	0	0	0	0	0	0	0	0
526	0	0	0	0	0	0	0	0	0
527	0	0	0	0	0	0	0	0	0
528	0	0	0	0	0	0	0	0	0

529	0	0	0	0	0	0	0	0	0
530	0	0	0	1	0	1	0	7	7
531	0	0	0	0	0	0	0	0	0
532	0	0	0	0	0	0	0	0	0
533	0	0	1	1	0	2	0	38	38
534	0	0	0	0	0	0	0	0	0
535	0	0	0	0	0	0	0	0	0
536	0	0	0	0	0	0	0	0	0
537	0	0	0	0	0	0	0	1	1
538	0	0	0	0	0	0	0	0	0
539	0	0	0	0	0	0	0	0	0
540	0	0	0	0	0	0	0	0	0
541	0	0	0	1	0	0	1	4	5
542	0	0	0	0	0	0	0	0	0
543	0	0	0	0	0	0	1	1	2
544	0	0	0	0	0	0	0	0	0
545	0	0	0	0	0	0	0	0	0
546	0	0	0	0	0	0	0	0	0
547	0	0	0	0	0	0	0	0	0
548	0	0	0	0	0	0	0	0	0
549	1	0	0	1	0	0	0	0	-1
550	0	0	0	0	0	0	0	0	0
551	0	2	0	1	0	0	1	2	1
552	0	0	0	2	0	2	0	37	37
553	0	0	0	0	0	0	0	0	0
554	0	0	0	1	0	0	0	14	14
555	0	0	0	0	0	0	0	0	0
556	0	0	0	0	0	0	0	0	0
557	0	0	0	0	0	0	0	0	0
558	0	0	0	0	0	0	0	0	0
559	1	1	0	0	0	0	1	0	-1
560	4	0	3	1	0	2	0	13	9
561	0	0	0	0	0	0	0	0	0
562	0	0	0	0	0	0	0	0	0
563	0	0	0	1	0	0	0	0	0
564	0	0	0	0	0	0	0	1	1
565	0	0	0	0	0	0	0	3	3
566	0	0	0	0	0	0	0	0	0
567	0	0	0	0	0	0	0	0	0
568	0	0	0	0	0	0	0	0	0
569	0	0	0	0	0	0	0	1	1
570	0	0	0	0	0	0	0	1	1
571	0	0	0	0	0	0	0	0	0
572	0	0	0	7	0	4	4	78	82
573	0	0	0	0	0	1	0	2	2
574	0	0	2	3	2	3	3	3	6
575	0	0	0	0	0	0	0	1	1
576	0	0	0	0	0	0	0	0	0
577	0	0	0	0	0	0	0	0	0
578	0	0	0	0	0	0	0	1	1
579	0	0	0	1	0	1	3	16	19
580	0	0	0	0	0	0	0	0	0
581	0	0	0	0	0	0	0	0	0

582	0	0	0	0	0	0	0	1	1
583	0	0	0	0	0	0	0	0	0
584	0	0	0	0	0	0	0	0	0
585	0	0	0	0	0	0	0	0	0
586	0	0	0	0	0	0	0	1	1
587	0	0	0	4	0	0	0	1	1
588	0	0	1	1	0	0	0	1	1
589	0	0	0	0	0	0	0	0	0
590	0	0	0	0	0	0	0	1	1
591	0	0	0	0	0	0	0	5	5
592	0	0	0	0	0	0	0	0	0
593	0	0	0	0	0	0	0	1	1
594	0	0	0	0	0	0	0	2	2
595	0	0	0	0	0	0	0	0	0
596	0	0	0	0	0	0	0	0	0
597	0	0	0	0	0	0	0	1	1
598	0	0	0	0	0	0	0	0	0
599	0	0	0	0	0	0	0	3	3
600	0	0	0	0	0	0	0	0	0
601	0	0	0	0	0	0	0	0	0
602	0	6	0	2	0	4	0	58	52
603	0	3	0	2	0	1	0	2	-1
604	0	0	0	1	2	2	0	2	2
605	0	0	0	0	0	0	0	0	0
606	0	0	0	0	0	0	0	0	0
607	0	0	0	0	0	0	0	0	0
608	0	0	0	0	0	0	0	0	0
609	0	0	0	0	0	0	0	0	0
610	0	0	0	0	0	0	0	1	1
611	0	0	0	0	0	0	0	3	3
612	0	0	0	0	0	0	0	2	2
613	0	0	0	0	0	0	0	0	0
614	0	0	0	0	0	0	0	0	0
615	0	0	0	0	0	0	0	0	0
616	0	0	0	0	0	0	0	0	0
617	0	2	0	0	0	0	0	1	-1
618	0	0	0	0	0	0	0	0	0
619	2	0	0	0	0	0	0	3	1
620	0	0	0	0	0	0	0	0	0
621	0	0	0	0	0	0	0	0	0
622	0	0	0	0	0	0	0	0	0
623	0	0	0	0	0	0	0	0	0
624	0	1	0	0	0	0	0	0	-1
625	0	0	0	0	0	0	1	5	6
626	0	0	0	0	0	0	0	0	0
627	0	0	0	0	0	0	0	0	0
628	0	0	0	0	0	0	0	0	0
629	0	0	0	0	0	0	0	1	1
630	0	1	0	0	1	1	1	0	0
631	0	0	0	0	0	0	0	0	0
632	0	4	0	3	0	4	1	60	57
633	0	0	0	0	0	0	0	0	0
634	0	2	0	0	0	0	0	11	9

635	0	0	0	0	0	0	0	0	0
636	0	0	0	0	0	0	0	11	11
637	0	0	0	0	0	0	0	0	0
638	0	0	0	1	0	0	0	1	1
639	0	0	0	0	0	0	0	0	0
640	0	0	0	0	0	0	0	0	0
641	0	0	0	0	0	0	0	5	5
642	0	0	0	0	0	0	0	0	0
643	0	0	0	0	0	0	0	13	13
644	0	0	0	0	0	0	0	2	2
645	0	0	0	0	0	0	1	25	26
646	0	0	0	0	0	0	0	2	2
647	0	0	0	0	0	0	0	0	0
648	0	0	0	1	0	0	1	1	2
649	0	0	0	0	0	0	0	0	0
650	0	0	0	0	0	0	0	0	0
651	0	0	0	0	0	0	0	0	0
652	0	0	0	1	0	0	0	0	0
653	0	0	0	0	0	0	0	0	0
654	0	0	0	0	0	0	0	0	0
655	0	0	0	0	0	0	0	0	0
656	0	0	0	0	0	0	0	0	0
657	0	0	0	0	0	0	0	4	4
658	0	0	0	0	0	0	0	2	2
659	0	0	0	0	0	0	0	0	0
660	0	0	0	0	0	0	0	1	1
661	0	0	0	0	0	0	0	0	0
662	0	0	0	0	0	0	0	0	0
663	0	0	0	0	0	0	0	0	0
664	0	0	0	0	0	0	0	0	0
665	0	0	0	0	0	0	0	0	0
666	0	0	0	0	0	0	0	0	0
667	0	0	0	0	0	0	0	0	0
668	0	0	0	0	0	0	0	0	0
669	0	0	0	0	0	0	0	0	0
670	0	0	0	0	0	0	0	0	0
671	0	0	0	0	0	0	0	0	0
672	0	1	0	0	0	0	0	0	-1
673	0	0	0	0	0	0	0	0	0
674	0	0	0	0	0	0	0	0	0
675	0	0	0	0	0	0	0	0	0
676	0	0	0	0	0	0	0	0	0
677	0	0	0	0	0	0	0	0	0
678	0	0	0	0	0	0	0	0	0
679	0	1	0	0	0	0	0	0	-1
680	0	0	0	0	0	0	0	0	0
681	0	0	0	0	0	0	1	3	4
682	0	0	0	0	0	0	0	0	0
683	0	0	0	0	0	0	0	12	12
684	0	0	0	0	0	0	0	0	0
685	0	0	0	0	0	0	0	0	0
686	0	0	0	0	0	0	0	0	0
687	0	0	0	0	0	0	0	0	0

688	0	0	0	0	0	0	0	0	0
689	0	0	0	0	0	0	0	0	0
690	0	0	0	0	0	0	0	0	0
691	0	0	0	0	0	0	0	0	0
692	0	0	0	0	0	0	0	0	0
693	0	0	0	0	0	0	0	0	0
694	0	1	0	0	0	0	0	2	1
695	0	0	0	1	0	0	1	1	2
696	0	0	0	0	0	0	0	0	0
697	0	0	0	0	0	2	0	8	8
698	0	3	0	0	0	1	0	7	4
699	0	0	0	0	0	0	0	0	0
700	0	0	0	0	0	0	0	0	0
701	0	0	1	0	0	1	0	5	5
702	0	0	0	0	0	0	0	0	0
703	0	1	0	0	0	0	0	1	0
704	0	0	0	0	0	0	0	0	0
705	0	0	0	0	0	0	0	0	0
706	0	0	0	0	0	0	0	1	1
707	0	0	0	0	0	0	0	0	0
708	0	0	0	0	0	0	0	0	0
709	0	0	0	0	0	0	0	0	0
710	1	2	0	0	0	0	1	1	-1
711	0	0	1	0	0	0	0	1	1
712	0	0	0	0	0	0	0	0	0
713	1	4	5	2	3	4	0	5	0
714	0	0	0	0	0	0	0	1	1
715	0	0	0	0	0	0	0	0	0
716	0	0	0	0	0	0	0	0	0
717	0	0	0	0	0	0	0	0	0
718	0	0	0	0	0	0	0	0	0
719	0	0	0	0	0	0	0	0	0
720	0	0	0	0	0	0	0	0	0
721	0	0	0	0	0	0	0	0	0
722	0	0	0	0	0	1	0	0	0
723	0	0	0	0	0	0	0	0	0
724	0	0	0	0	0	0	0	0	0
725	0	0	0	0	0	0	0	0	0
726	0	0	0	0	0	0	0	0	0
727	0	0	0	0	0	0	0	0	0
728	0	0	0	0	0	0	0	0	0
729	0	0	0	0	0	0	0	0	0
730	0	0	0	0	0	0	0	0	0
731	0	0	0	0	0	0	0	1	1
732	0	0	0	0	0	0	0	0	0
733	0	0	0	0	0	0	0	0	0
734	0	0	0	0	0	0	0	0	0
735	0	0	0	0	0	0	0	0	0
736	0	0	0	0	0	0	0	0	0
737	0	0	0	0	0	0	0	0	0
738	0	0	0	0	0	0	0	0	0
739	0	0	0	0	0	0	0	0	0
740	0	0	0	0	0	0	0	0	0

741	0	0	0	0	0	0	0	0	0
742	0	0	1	0	0	0	0	0	0
743	0	0	0	0	0	0	0	0	0
744	0	0	0	0	0	0	0	0	0
745	0	0	0	0	0	0	0	0	0
746	0	0	0	0	0	0	0	0	0
747	0	0	0	0	0	0	0	0	0
748	0	0	0	0	0	0	0	0	0
749	0	0	0	0	0	0	0	0	0
750	0	0	0	0	0	0	0	0	0
751	0	0	0	0	0	0	0	1	1
752	0	0	0	0	0	0	0	0	0
753	0	0	0	0	0	0	1	1	2
754	1	0	0	0	0	0	0	0	-1
755	0	0	0	0	0	0	0	0	0
756	0	0	0	2	0	1	3	9	12
757	3	9	9	0	17	2	1	3	-8
758	0	0	0	0	0	0	0	1	1
759	0	0	0	0	0	0	0	1	1
760	0	0	0	0	0	0	0	0	0
761	0	0	0	1	0	0	0	3	3
762	0	0	0	0	0	0	0	0	0
763	0	0	0	0	0	0	0	0	0
764	0	0	0	0	0	0	0	0	0
765	0	2	1	0	1	1	1	2	1
766	1	0	3	0	0	2	3	4	6
767	0	0	0	0	0	0	0	0	0
768	0	0	0	0	0	0	0	0	0
769	0	1	0	0	2	1	1	2	2
770	0	3	0	0	1	0	3	1	1
771	0	0	0	0	0	0	0	1	1
772	0	0	0	0	0	0	0	0	0
773	0	0	0	0	0	0	0	0	0
774	0	0	0	0	0	0	0	0	0
775	0	0	0	0	0	0	0	0	0
776	0	0	0	0	0	0	0	0	0
777	0	0	0	0	0	0	0	0	0
778	0	0	0	0	0	0	0	0	0
779	0	0	0	0	0	0	0	0	0
780	0	12	0	1	0	2	0	0	-12
781	0	0	0	0	0	0	0	0	0
782	0	0	0	0	0	0	0	0	0
783	0	0	0	0	0	0	0	0	0
784	0	0	0	0	0	0	0	0	0
785	0	0	0	0	0	0	0	0	0
786	0	0	0	0	0	0	0	0	0
787	0	3	0	0	0	0	0	0	-3
788	0	0	0	0	0	0	0	0	0
789	0	0	0	0	0	0	0	0	0
790	0	0	0	0	0	0	0	0	0
791	0	0	0	0	0	0	0	0	0
792	0	0	0	0	0	0	0	0	0
793	0	0	0	0	0	0	0	0	0

794	0	0	0	0	0	0	0	0	0
795	0	0	0	0	0	0	0	0	0
796	0	0	1	0	0	0	0	0	0
797	0	0	0	0	0	0	0	0	0
798	0	0	0	0	0	0	0	0	0
799	0	0	0	0	0	0	0	0	0
800	0	0	1	0	0	0	0	0	0
801	1	12	1	2	1	0	2	7	-4
802	0	0	0	0	0	0	0	0	0
803	1	0	0	0	0	0	0	0	-1
804	0	0	0	0	0	0	0	0	0
805	0	0	0	0	0	0	0	1	1
806	0	0	0	0	0	0	0	0	0
807	0	0	0	0	0	0	0	0	0
808	0	0	0	0	0	0	0	0	0
809	0	0	0	0	0	0	0	0	0
810	0	0	0	0	0	0	0	0	0
811	0	0	0	0	0	0	0	0	0
812	0	0	0	0	0	0	0	0	0
813	0	0	0	0	0	0	0	0	0
814	0	0	0	0	0	0	0	0	0
815	0	0	0	0	0	0	0	0	0
816	0	0	0	0	0	0	0	0	0
817	0	0	0	0	0	0	0	0	0
818	0	0	0	0	0	0	1	0	1
819	0	0	0	0	0	0	0	0	0
820	0	0	0	0	0	0	0	0	0
821	0	0	0	0	0	0	0	0	0
822	0	0	0	0	0	0	0	0	0
823	0	0	0	0	0	0	0	0	0
824	0	0	0	0	0	0	0	0	0
825	0	0	0	0	0	0	0	0	0
826	0	0	0	0	0	0	0	0	0
827	0	0	0	0	0	0	0	0	0
828	0	0	0	0	0	0	0	0	0
829	0	0	0	0	0	0	0	1	1
830	0	0	0	0	0	0	0	0	0
831	0	0	0	0	0	0	0	1	1
832	0	0	0	0	0	0	0	0	0
833	0	0	0	0	0	0	0	0	0
834	0	0	0	0	0	0	0	0	0
835	0	0	0	0	0	0	0	0	0
836	0	0	0	0	0	0	0	0	0
837	0	0	0	0	0	0	0	0	0
838	0	0	0	0	0	0	0	0	0
839	0	0	0	0	0	0	0	0	0
840	0	0	0	0	0	0	0	0	0
841	0	0	0	0	0	0	0	0	0
842	0	0	0	0	0	0	0	0	0
843	0	0	0	0	0	0	0	0	0
844	0	0	0	0	0	0	0	0	0
845	0	0	0	0	0	0	0	0	0
846	0	0	0	0	0	0	0	0	0

847	0	0	0	0	0	1	0	0	0
848	0	0	0	0	0	0	0	0	0
849	0	0	0	0	0	0	0	0	0
850	0	0	0	0	0	0	0	0	0
851	0	0	0	0	0	0	0	0	0
852	0	0	0	0	0	0	0	0	0
853	0	0	0	0	0	0	0	0	0
854	0	0	0	0	0	0	0	0	0
855	0	0	0	0	0	0	0	0	0
856	0	0	0	0	0	0	0	0	0
857	0	0	2	0	0	0	0	3	3
858	0	0	0	0	0	0	0	0	0
859	0	0	0	0	0	0	0	0	0
860	0	0	0	0	0	0	0	0	0
861	0	0	0	0	0	0	0	0	0
862	0	0	0	0	0	0	0	0	0
863	0	0	0	0	0	0	0	0	0
864	0	0	0	0	0	0	0	0	0
865	0	0	0	0	0	0	0	1	1
866	0	0	0	0	0	0	0	0	0
867	0	0	0	0	0	0	0	1	1
868	0	0	0	0	0	0	0	0	0
869	0	0	0	0	0	0	0	1	1
870	0	0	0	0	0	0	0	0	0
871	0	0	0	0	0	0	0	0	0
872	0	0	0	0	0	0	0	0	0
873	0	0	0	0	0	0	0	0	0
874	0	0	0	0	0	0	0	0	0
875	0	0	0	0	0	0	0	0	0
876	0	0	2	0	0	1	0	3	3
877	0	0	0	0	0	0	0	0	0
878	0	0	0	0	0	0	0	0	0
879	0	0	0	0	0	0	0	0	0
880	0	0	1	0	0	0	0	0	0
881	0	0	0	0	0	0	0	1	1
882	0	0	0	0	0	2	0	2	2
883	0	0	0	0	0	0	0	0	0
884	0	0	0	0	0	0	0	0	0
885	0	0	0	0	0	0	0	0	0
886	0	0	0	0	0	0	0	0	0
887	0	0	0	0	0	0	0	0	0
888	0	0	0	0	0	0	0	0	0
889	0	0	0	0	0	0	0	0	0
890	0	0	0	0	0	0	0	1	1
891	0	0	0	0	0	0	0	0	0
892	0	0	0	0	0	0	0	0	0
893	0	0	0	0	0	0	0	0	0
894	0	0	0	0	0	0	0	0	0
895	0	0	0	0	0	0	0	0	0
896	0	0	0	0	0	0	0	0	0
897	0	1	0	0	0	0	0	1	0
898	0	0	0	0	0	0	0	0	0
899	0	0	0	0	0	0	0	1	1

900	0	0	0	0	0	1	0	0	0
901	0	0	0	0	0	0	0	1	1
902	0	0	0	0	0	0	0	0	0
903	0	0	0	0	0	0	0	0	0
904	0	0	0	0	0	0	0	0	0
905	0	0	0	0	0	0	0	0	0
906	0	0	0	0	0	0	0	0	0
907	0	0	0	0	0	0	0	0	0
908	0	0	0	0	0	0	0	0	0
909	0	0	1	0	0	0	0	0	0
910	0	0	0	0	0	0	0	0	0
911	0	0	0	0	0	0	0	0	0
912	0	0	0	0	0	0	0	0	0
913	0	0	0	0	0	0	0	0	0
914	0	0	0	0	0	0	0	0	0
915	0	0	0	0	0	0	0	0	0
916	0	0	0	0	0	0	0	0	0
917	0	0	0	0	0	0	0	0	0
918	0	0	0	0	0	0	0	0	0
919	0	2	0	0	0	0	0	0	-2
920	0	0	0	0	0	0	0	0	0
921	0	0	0	0	0	0	0	1	1
922	0	0	0	0	0	0	0	0	0
923	0	0	0	0	0	0	0	0	0
924	0	0	0	0	0	0	0	0	0
925	0	0	0	0	0	0	0	1	1
926	0	0	0	0	0	0	0	0	0
927	0	0	0	0	0	0	0	0	0
928	0	0	0	0	0	0	0	0	0
929	0	0	0	0	0	0	0	0	0
930	0	0	0	0	0	0	0	0	0
931	0	0	0	0	0	0	0	0	0
932	0	0	0	0	0	0	0	0	0
933	0	0	0	0	0	0	0	0	0
934	1	4	0	0	0	0	0	0	-5
935	0	1	0	0	0	0	0	0	-1
936	0	0	0	0	0	0	0	0	0
937	0	0	0	0	0	0	0	0	0
938	0	0	0	0	0	0	0	1	1
939	0	0	0	0	0	0	0	0	0
940	0	0	0	0	0	0	0	0	0
941	0	0	0	0	0	0	0	0	0
942	0	0	0	0	0	0	0	0	0
943	0	0	0	0	0	0	0	0	0
944	0	0	0	0	0	0	0	0	0
945	0	0	0	0	0	0	0	0	0
946	0	0	0	0	0	0	0	0	0
947	0	0	0	0	0	0	0	0	0
948	0	0	1	0	0	1	0	4	4
949	0	0	0	0	0	0	0	0	0
950	0	0	0	0	0	0	0	0	0
951	0	0	0	0	0	0	0	0	0
952	0	0	0	0	0	0	0	0	0

953	0	0	2	0	2	0	0	0	0
954	1	0	0	0	0	0	0	0	-1
955	0	0	0	0	0	0	0	0	0
956	0	0	1	0	0	0	0	0	0
957	0	0	0	0	0	0	0	0	0
958	0	0	0	0	0	0	0	0	0
959	0	0	0	0	0	0	0	0	0
960	0	0	0	0	0	0	0	0	0
961	0	0	0	0	0	0	0	0	0
962	0	0	0	0	0	0	0	0	0
963	0	0	0	0	0	0	0	0	0
964	0	0	0	0	0	0	0	0	0
965	0	0	0	0	0	0	0	0	0
966	0	0	0	0	0	0	0	0	0
967	0	0	0	0	0	0	0	0	0
968	0	0	0	0	0	0	0	0	0
969	0	0	0	0	0	0	0	0	0
970	0	0	0	0	0	0	0	0	0
971	0	0	0	0	0	0	0	0	0
972	0	0	0	0	0	0	0	0	0
973	0	0	0	0	0	0	0	0	0
974	0	0	0	0	0	0	0	0	0
975	0	0	0	0	0	0	0	4	4
976	0	0	0	0	0	0	0	0	0
977	0	0	0	0	0	0	0	0	0
978	0	0	0	0	0	0	0	0	0
979	0	0	0	0	0	0	0	0	0
980	0	0	0	0	0	0	0	0	0
981	1	4	3	4	1	1	0	2	-3
982	0	1	0	0	0	1	0	5	4
983	0	0	0	0	0	0	0	0	0
984	2	0	2	1	0	4	0	7	5
985	0	0	0	0	0	0	0	0	0
986	0	0	0	0	0	0	0	0	0
987	0	0	0	0	0	0	0	0	0
988	0	0	3	0	0	0	0	0	0
989	0	0	0	0	0	0	0	0	0
990	0	0	0	0	0	0	0	0	0
991	0	0	0	0	0	0	0	0	0
992	0	0	0	0	0	0	0	0	0
993	0	1	0	0	0	0	0	0	-1
994	1	3	1	2	4	2	0	0	-4
995	0	0	0	0	0	0	0	0	0
996	0	0	0	0	0	0	0	0	0
997	0	0	0	0	0	0	0	0	0
998	0	0	0	0	0	0	0	0	0
999	0	0	0	0	0	0	0	0	0
1000	0	0	0	0	0	0	0	0	0
1001	0	0	0	0	0	0	0	0	0
1002	0	0	0	0	0	0	0	0	0
1003	0	0	0	0	0	0	0	0	0
1004	0	0	0	0	0	0	0	0	0
1005	0	0	0	0	0	0	0	0	0

1006	0	0	0	0	0	0	0	1	1
1007	0	0	0	0	0	0	0	0	0
1008	0	0	0	0	0	0	0	2	2
1009	0	0	0	0	0	0	0	0	0
1010	0	0	0	0	0	0	0	0	0
1011	1	0	0	1	0	0	0	0	-1
1012	0	0	0	0	0	0	0	0	0
1013	0	0	0	0	0	0	0	1	1
1014	0	0	2	0	0	1	0	1	1
1015	1	0	0	0	0	1	1	2	2
1016	0	0	0	0	0	0	0	2	2
1017	0	0	0	0	0	0	0	0	0
1018	0	0	0	0	0	0	0	0	0
1019	0	0	0	0	0	0	0	2	2
1020	0	1	0	0	0	0	0	0	-1
1021	0	0	0	0	0	0	0	0	0
1022	0	0	0	0	0	0	0	0	0
1023	0	0	0	0	0	0	0	0	0
1024	0	0	0	0	0	0	0	0	0
1025	0	0	0	0	0	0	0	2	2
1026	0	0	0	0	0	0	0	0	0
1027	0	0	1	0	0	0	0	0	0
1028	0	0	0	0	0	0	0	1	1
1029	0	0	0	0	0	0	0	0	0
1030	2	0	0	0	0	0	0	1	-1
1031	0	0	0	0	0	0	0	1	1
1032	0	0	0	0	0	0	0	0	0
1033	0	0	0	0	0	0	1	0	1
1034	0	0	0	0	0	0	0	0	0
1035	0	0	0	0	0	0	0	0	0
1036	0	0	0	0	0	0	0	0	0
1037	0	0	0	0	0	0	0	1	1
1038	0	0	0	0	0	0	0	1	1
1039	0	0	0	0	0	0	0	0	0
1040	0	0	0	0	0	0	0	0	0
1041	0	0	0	0	0	0	0	0	0
1042	0	0	0	0	0	0	0	0	0
1043	0	0	0	0	0	0	0	0	0
1044	0	0	0	0	0	0	0	0	0
1045	0	0	0	0	1	0	0	0	0
1046	0	0	0	0	0	0	0	0	0
1047	0	0	0	0	0	0	0	0	0
1048	2	0	0	0	0	0	0	0	-2
1049	0	0	0	0	0	0	0	0	0
1050	0	0	0	0	0	0	0	0	0
1051	0	0	0	0	0	0	0	0	0
1052	1	0	0	0	0	0	0	0	-1
1053	1	0	0	0	0	0	0	0	-1
1054	1	0	0	0	0	0	0	0	-1
1055	0	0	0	0	0	0	0	0	0
1056	0	0	0	0	0	0	0	0	0
1057	0	0	0	0	0	0	0	0	0
1058	0	0	0	0	0	0	0	0	0

1059	0	0	0	0	0	0	0	0	0
1060	0	0	0	0	0	0	0	0	0
1061	0	0	0	1	0	0	0	0	0
1062	0	1	0	0	0	0	0	0	-1
1063	0	0	0	0	0	0	0	0	0
1064	0	0	0	0	0	0	0	0	0
1065	0	0	0	1	0	1	0	0	0
1066	0	0	0	0	0	0	0	0	0
1067	0	0	0	0	0	0	0	0	0
1068	0	0	0	0	0	0	0	0	0
1069	0	0	1	0	1	1	0	1	1
1070	0	0	0	0	0	0	0	0	0
1071	0	0	0	0	0	0	0	0	0
1072	0	0	0	0	0	0	0	0	0
1073	1	0	0	0	0	0	0	0	-1
1074	0	0	0	0	0	1	1	0	1
1075	0	0	0	0	0	0	0	0	0
1076	0	0	0	0	0	0	0	0	0
1077	0	0	0	0	0	0	0	0	0
1078	0	0	0	0	0	0	0	0	0
1079	0	0	0	0	0	0	0	0	0
1080	0	0	0	0	0	0	0	1	1
1081	0	0	0	0	0	0	0	0	0
1082	0	0	0	0	0	0	0	0	0
1083	0	0	0	0	0	0	0	0	0
1084	0	0	0	0	0	0	0	0	0
1085	0	0	0	0	0	0	0	0	0
1086	0	0	0	4	1	1	0	2	2
1087	0	0	0	0	0	0	0	0	0
1088	0	0	0	0	0	0	0	0	0
1089	1	0	0	0	0	0	0	0	-1
1090	0	0	0	0	0	0	0	1	1
1091	0	0	0	0	0	0	0	0	0
1092	0	0	0	0	0	0	0	0	0
1093	0	0	0	0	0	0	0	0	0
1094	0	0	0	0	0	0	0	0	0
1095	1	0	0	0	0	0	0	0	-1
1096	0	0	0	0	0	0	0	0	0
1097	0	0	0	0	0	0	0	0	0
1098	0	0	0	0	0	0	0	0	0
1099	0	0	0	0	0	0	0	0	0
1100	0	0	0	0	0	0	0	0	0
1101	0	0	0	0	0	0	0	0	0
1102	0	0	0	0	0	0	0	0	0
1103	0	0	0	0	0	0	0	0	0
1104	0	0	0	0	0	0	0	0	0
1105	0	0	0	0	0	0	0	0	0
1106	0	0	0	0	0	0	0	0	0
1107	0	0	0	0	0	0	0	0	0
1108	0	0	0	0	0	0	0	0	0
1109	0	0	0	0	0	0	0	0	0
1110	0	0	0	0	0	0	0	0	0
1111	0	0	0	0	0	0	0	0	0

1112	0	0	0	0	0	0	0	0	0
1113	0	0	0	0	0	0	0	1	1
1114	0	0	0	0	0	0	0	0	0
1115	0	0	0	0	0	0	0	0	0
1116	0	0	0	0	0	0	0	0	0
1117	0	0	0	0	0	0	0	0	0
1118	0	0	0	0	0	0	0	0	0
1119	0	0	0	0	0	0	0	0	0
1120	0	0	0	0	0	0	0	0	0
1121	0	0	0	0	0	0	0	0	0
1122	0	0	0	0	0	0	0	0	0
1123	0	0	0	0	0	0	0	0	0
1124	0	0	0	0	0	0	0	0	0
1125	0	0	0	0	0	0	0	0	0
1126	0	0	0	0	0	0	0	1	1
1127	0	0	0	0	0	0	0	0	0
1128	0	0	0	0	0	0	0	0	0
1129	0	0	0	0	0	0	0	0	0
1130	0	1	3	0	0	2	0	1	0
1131	0	0	0	0	0	0	0	0	0
1132	0	0	0	0	0	0	0	0	0
1133	0	0	0	0	0	0	0	0	0
1134	0	0	0	0	0	0	0	0	0
1135	0	0	0	0	0	0	0	0	0
1136	0	0	0	0	0	0	0	0	0
1137	0	0	0	0	0	0	0	2	2
1138	0	0	0	0	0	0	0	0	0
1139	0	0	0	0	0	0	0	0	0
1140	0	0	0	0	0	0	0	2	2
1141	0	0	0	0	0	0	0	0	0
1142	0	0	0	0	0	0	0	0	0
1143	0	0	0	0	0	0	0	0	0
1144	0	0	0	0	0	0	0	0	0
1145	0	0	0	0	0	0	0	0	0
1146	0	0	0	0	0	0	0	0	0
1147	0	0	0	0	0	0	0	0	0
1148	0	7	0	0	5	0	0	0	-7
1149	0	0	0	0	0	0	0	0	0
1150	0	0	0	0	0	0	0	0	0
1151	0	0	0	0	0	0	0	0	0
1152	0	1	2	0	0	0	0	0	-1
1153	0	1	0	0	0	0	0	0	-1
1154	0	1	2	0	0	0	0	0	-1
1155	0	2	0	0	0	0	0	0	-2
1156	0	0	0	0	0	0	0	0	0
1157	0	0	0	0	0	0	0	0	0
1158	0	0	0	0	0	0	0	0	0
1159	0	0	0	0	0	0	0	2	2
1160	0	0	0	0	0	0	0	0	0
1161	0	0	0	0	0	0	0	0	0
1162	0	0	0	0	0	0	0	0	0
1163	0	0	0	0	0	0	0	0	0
1164	0	0	0	0	0	0	0	0	0

1165	0	0	0	0	0	0	0	0	0
1166	0	0	0	0	0	0	0	0	0
1167	0	0	0	0	0	0	0	0	0
1168	0	0	0	0	0	0	0	0	0
1169	0	0	0	0	0	0	0	1	1
1170	0	0	0	0	0	0	0	0	0
1171	0	0	0	0	0	0	0	1	1
1172	0	0	0	0	0	0	0	1	1
1173	0	0	1	0	0	1	0	0	0
1174	0	0	0	0	0	0	0	0	0
1175	0	0	0	0	0	0	0	0	0
1176	0	0	0	0	0	0	0	3	3
1177	0	0	0	0	0	0	0	0	0
1178	0	0	0	0	0	0	0	0	0
1179	0	0	0	0	0	0	0	1	1
1180	0	0	0	0	0	0	0	1	1
1181	0	0	0	0	0	0	0	0	0
1182	0	0	0	0	0	0	0	0	0
1183	0	0	0	0	0	0	0	0	0
1184	0	0	0	0	0	0	0	0	0
1185	0	0	0	0	0	0	0	0	0
1186	0	0	0	0	0	0	0	0	0
1187	0	0	0	0	0	0	0	0	0
1188	0	0	0	0	0	0	0	0	0
1189	0	0	0	0	0	0	0	0	0
1190	0	0	0	0	0	0	0	0	0
1191	0	0	0	0	0	0	0	1	1
1192	0	0	0	0	0	0	0	2	2
1193	0	1	0	0	0	0	0	2	1
1194	0	0	0	0	0	0	0	0	0
1195	0	1	0	0	0	0	0	0	-1
1196	0	1	0	0	0	0	0	1	0
1197	0	0	0	0	0	0	0	2	2
1198	0	0	0	0	0	0	0	0	0
1199	0	0	0	0	0	0	0	0	0
1200	0	0	0	0	0	0	0	0	0
1201	0	0	1	0	0	1	1	7	8
1202	0	0	0	0	0	0	0	0	0
1203	0	0	0	0	0	0	0	0	0
1204	0	0	0	0	0	0	0	0	0
1205	0	0	0	0	0	0	0	0	0
1206	0	0	0	0	0	0	0	0	0
1207	0	0	0	0	0	0	0	1	1
1208	0	0	0	0	0	0	0	0	0
1209	1	1	0	0	0	0	0	0	-2
1210	0	0	0	0	0	0	0	0	0
1211	0	0	0	0	0	0	0	0	0
1212	0	0	0	0	0	0	0	2	2
1213	0	0	0	0	0	0	0	0	0
1214	0	0	0	0	0	0	0	0	0
1215	0	0	0	0	0	0	0	0	0
1216	0	0	0	0	0	0	0	0	0
1217	0	0	0	0	0	0	0	0	0

1218	0	0	0	0	0	0	0	0	0
1219	0	0	0	0	0	0	0	0	0
1220	0	0	0	0	0	0	0	0	0
1221	0	0	0	0	0	0	0	1	1
1222	0	0	0	0	0	0	0	0	0
1223	0	0	0	0	0	0	0	0	0
1224	0	0	0	0	0	0	0	0	0
1225	0	0	0	0	0	0	0	0	0
1226	0	0	0	0	0	0	0	0	0
1227	0	0	0	0	0	0	0	0	0
1228	0	0	0	0	0	0	0	0	0
1229	0	0	0	0	0	0	0	0	0
1230	0	0	0	0	0	0	0	0	0
1231	0	0	0	0	0	0	0	0	0
1232	0	0	0	0	0	0	0	0	0
1233	0	0	0	0	0	0	0	0	0
1234	0	0	0	0	0	0	0	0	0
1235	0	0	0	0	0	0	0	0	0
1236	0	0	0	0	0	0	0	1	1
1237	0	0	0	0	0	0	0	0	0
1238	0	0	0	0	0	0	0	0	0
1239	0	0	0	0	0	0	0	0	0
1240	0	0	0	0	0	0	0	0	0
1241	1	4	0	0	0	0	0	0	-5
1242	0	0	0	0	0	0	0	0	0
1243	0	1	0	0	0	0	1	1	1
1244	0	0	0	0	0	0	0	0	0
1245	0	1	0	0	0	0	0	0	-1
1246	0	0	0	0	0	0	0	0	0
1247	0	0	0	0	0	0	0	0	0
1248	0	7	4	0	9	0	0	0	-7
1249	0	0	0	0	0	0	0	1	1
1250	0	0	0	0	0	0	0	0	0
1251	0	0	0	0	0	0	0	0	0
1252	1	3	0	0	0	0	0	0	-4
1253	0	1	0	0	0	0	0	0	-1
1254	0	1	0	0	0	0	0	0	-1
1255	0	0	0	0	0	0	0	0	0
1256	0	0	0	0	0	0	0	1	1
1257	0	0	0	0	0	0	0	6	6
1258	0	0	0	0	0	0	0	0	0
1259	0	0	0	0	0	0	0	0	0
1260	0	0	0	0	0	0	0	0	0
1261	3	6	4	0	9	2	0	3	-6
1262	0	0	0	0	0	0	0	0	0
1263	0	0	0	0	0	0	0	0	0
1264	0	0	0	0	0	0	0	0	0
1265	0	0	0	0	0	0	0	0	0
1266	0	0	0	0	0	0	0	0	0
1267	0	0	0	0	0	0	0	0	0
1268	0	0	0	0	0	0	0	0	0
1269	0	0	0	0	0	0	0	0	0
1270	0	0	0	0	0	0	0	2	2

1271	0	0	0	0	0	0	0	1	1
1272	0	0	0	0	0	0	0	0	0
1273	0	1	0	0	0	0	0	0	-1
1274	0	0	0	0	0	0	0	0	0
1275	0	0	0	0	0	0	0	0	0
1276	0	0	0	0	0	0	0	0	0
1277	0	0	0	0	0	0	0	0	0
1278	0	0	0	0	0	0	0	0	0
1279	0	0	0	0	0	0	0	0	0
1280	0	0	0	0	0	0	0	0	0
1281	0	0	0	0	0	0	0	0	0
1282	0	0	0	0	0	0	0	1	1
1283	0	0	0	1	0	0	0	1	1
1284	0	0	0	0	0	0	0	1	1
1285	0	0	0	0	0	0	0	2	2
1286	0	0	0	0	0	0	0	0	0
1287	2	0	0	0	0	0	0	0	-2
1288	0	0	0	0	0	0	0	0	0
1289	0	0	0	0	0	0	0	0	0
1290	0	2	0	0	0	0	0	0	-2
1291	1	1	3	0	4	1	0	1	-1
1292	0	0	0	0	0	0	0	0	0
1293	0	0	0	0	0	0	0	0	0
1294	0	0	0	0	0	0	0	1	1
1295	0	0	0	0	0	0	0	0	0
1296	0	0	0	0	0	0	0	0	0
1297	0	0	0	0	0	0	0	0	0
1298	0	0	0	0	0	0	0	0	0
1299	0	0	0	0	0	0	0	1	1
1300	0	0	0	0	0	0	0	8	8
1301	0	0	0	0	0	0	0	1	1
1302	0	0	0	0	0	0	0	0	0
1303	0	0	0	0	0	0	0	0	0
1304	0	0	0	0	0	0	0	0	0
1305	0	0	0	0	0	0	0	0	0
1306	0	0	0	0	0	0	0	0	0
1307	0	0	0	0	0	0	0	0	0
1308	0	0	0	0	0	0	0	0	0
1309	0	0	0	0	0	0	0	0	0
1310	0	2	0	0	0	0	0	0	-2
1311	0	0	0	0	0	0	0	0	0
1312	0	0	0	0	0	0	0	4	4
1313	0	0	0	0	0	0	0	2	2
1314	0	1	0	0	0	0	0	0	-1
1315	0	0	0	0	0	0	0	0	0
1316	0	0	0	0	0	0	0	0	0
1317	0	0	0	0	0	0	0	0	0
1318	0	0	0	0	0	0	0	0	0
1319	0	0	0	0	0	0	0	0	0
1320	0	0	0	0	0	0	0	0	0
1321	0	0	0	0	0	0	0	0	0
1322	0	0	0	0	0	0	0	0	0
1323	0	0	0	0	0	0	0	0	0

1324	0	0	0	0	0	0	0	1	1
1325	0	0	0	0	0	0	0	0	0
1326	0	0	0	0	0	0	0	0	0
1327	0	0	0	0	0	0	0	0	0
1328	0	0	0	0	0	0	0	0	0
1329	0	0	0	0	0	0	0	0	0
1330	0	0	0	0	0	0	0	0	0
1331	0	0	0	0	0	0	0	0	0
1332	0	0	0	0	0	0	0	0	0
1333	0	0	0	0	0	0	0	0	0
1334	0	0	0	0	0	0	0	0	0
1335	0	0	0	0	0	0	0	0	0
1336	0	0	0	0	0	0	0	0	0
1337	0	0	0	0	0	0	0	1	1
1338	0	0	0	0	0	0	0	0	0
1339	0	0	0	0	0	0	0	0	0
1340	0	0	0	0	0	0	0	0	0
1341	0	0	0	0	0	0	0	0	0
1342	0	0	0	0	0	0	0	0	0
1343	0	0	0	0	0	0	0	0	0
1344	0	0	0	0	0	0	0	0	0
1345	0	0	0	0	0	0	0	0	0
1346	0	0	0	0	0	0	0	0	0
1347	0	0	0	0	0	0	0	0	0
1348	0	0	0	0	0	0	0	0	0
1349	0	0	0	0	0	0	0	0	0
1350	0	0	0	0	0	0	0	0	0
1351	0	0	0	0	0	0	0	0	0
1352	0	0	0	0	0	0	0	0	0
1353	0	0	0	0	0	0	0	0	0
1354	0	0	0	0	0	0	0	0	0
1355	0	0	0	0	0	0	0	0	0
1356	0	0	0	0	0	0	0	0	0
1357	0	0	0	0	0	0	0	0	0
1358	0	0	0	0	0	0	0	0	0
1359	0	0	0	0	0	0	0	0	0
1360	0	0	0	0	0	0	0	0	0
1361	0	0	0	0	0	0	0	0	0
1362	0	0	0	0	0	0	0	0	0
1363	0	0	0	0	0	0	0	0	0
1364	0	0	0	0	0	0	0	0	0
1365	0	0	0	0	0	0	0	0	0
1366	0	0	0	0	0	0	0	3	3
1367	0	0	0	0	0	0	0	0	0
1368	0	0	0	0	0	0	0	0	0
1369	0	0	0	0	0	0	0	0	0
1370	0	0	0	0	0	0	0	0	0
1371	0	0	0	0	0	0	0	0	0
1372	0	0	0	0	0	0	0	0	0
1373	0	0	0	0	0	0	0	0	0
1374	0	0	0	0	0	0	0	0	0
1375	0	0	0	0	0	0	0	0	0
1376	0	0	0	0	0	0	0	0	0

1377	0	0	0	0	0	0	0	0	0
1378	0	0	0	0	0	0	0	0	0
1379	0	0	0	0	0	0	0	0	0
1380	0	0	0	0	0	0	0	0	0
1381	0	0	0	0	0	0	0	0	0
1382	0	0	0	0	0	0	0	0	0
1383	0	0	0	0	0	0	0	0	0
1384	0	0	0	0	0	0	0	0	0
1385	0	0	0	0	0	0	0	0	0
1386	0	0	0	0	0	0	0	0	0
1387	0	0	0	0	0	0	0	0	0
1388	0	0	0	0	0	0	0	0	0
1389	0	0	0	0	0	0	0	0	0
1390	0	0	0	0	0	0	0	0	0
1391	0	0	0	0	0	0	0	0	0
1392	0	0	0	0	0	0	0	0	0
1393	0	0	0	0	0	0	0	0	0
1394	0	0	0	0	0	0	0	0	0
1395	0	0	0	0	0	0	0	0	0
1396	0	0	0	0	0	0	0	0	0
1397	0	0	0	0	0	0	0	0	0
1398	0	0	0	0	0	0	0	0	0
1399	0	0	0	0	0	0	0	0	0
1400	0	0	0	0	0	0	0	0	0
1401	0	0	0	0	0	0	0	0	0
1402	0	0	0	0	0	0	0	0	0
1403	0	0	0	0	0	0	0	0	0
1404	0	0	0	0	0	0	0	0	0
1405	0	0	0	0	0	0	0	0	0
1406	0	0	0	0	0	0	0	0	0
1407	0	0	0	0	0	0	0	0	0
1408	0	0	0	0	0	0	0	0	0
1409	0	0	0	0	0	0	0	2	2
1410	0	0	0	0	0	0	0	0	0
1411	0	0	0	0	0	0	0	0	0
1412	0	0	0	0	0	0	0	2	2
1413	0	0	0	0	0	0	0	0	0
1414	0	0	0	0	0	0	0	0	0
1415	0	0	0	0	0	0	0	0	0
1416	0	0	0	0	0	0	0	0	0
1417	0	0	0	0	0	0	0	0	0
1418	0	0	0	0	0	0	0	0	0
1419	0	0	0	0	0	0	0	0	0
1420	0	0	0	0	0	0	0	0	0
1421	0	0	0	0	0	0	0	0	0
1422	0	0	0	0	0	0	0	0	0
1423	0	0	0	0	0	0	0	0	0
1424	0	0	0	0	0	0	0	0	0
1425	0	0	0	0	0	0	0	0	0
1426	0	0	0	0	0	0	0	0	0
1427	0	0	0	0	0	0	0	0	0
1428	0	0	0	0	0	0	0	0	0
1429	0	0	0	0	0	0	0	0	0

1430	0	0	0	0	0	0	0	0	0
1431	0	0	0	0	0	0	0	0	0
1432	0	0	0	1	0	2	0	7	7
1433	0	0	0	0	0	0	0	0	0
1434	0	0	0	0	0	0	0	0	0
1435	0	0	0	0	0	0	0	0	0
1436	0	0	0	0	0	0	0	0	0
1437	0	0	0	0	0	0	0	0	0
1438	0	0	0	0	0	0	0	0	0
1439	0	0	0	0	0	0	0	0	0
1440	0	0	0	0	0	0	0	0	0
1441	0	0	0	0	0	0	0	0	0
1442	0	0	0	0	0	0	0	0	0
1443	0	0	0	0	0	0	0	0	0
1444	0	0	0	0	0	0	0	1	1
1445	0	0	0	0	0	0	0	0	0
1446	0	0	0	0	0	0	0	0	0
1447	0	0	0	0	0	0	0	0	0
1448	0	0	0	0	0	0	0	0	0
1449	0	0	0	0	0	0	0	0	0
1450	0	0	0	0	0	0	0	0	0
1451	0	0	0	0	0	0	0	0	0
1452	0	0	0	0	0	0	0	0	0
1453	0	0	0	0	0	0	0	0	0
1454	0	0	0	0	0	0	0	0	0
1455	0	0	0	0	0	0	0	0	0
1456	0	0	0	0	0	0	0	0	0
1457	0	0	0	0	0	0	0	0	0
1458	0	0	0	0	0	0	0	0	0
1459	0	0	0	0	0	0	0	0	0
1460	0	0	0	0	0	0	0	0	0
1461	0	0	0	0	0	0	0	0	0
1462	0	0	0	0	0	0	0	0	0
1463	0	0	0	0	0	0	0	0	0
1464	0	0	0	0	0	0	0	0	0
1465	0	0	0	0	0	0	0	0	0
1466	0	0	0	0	0	0	0	0	0
1467	0	0	0	0	0	0	0	0	0
1468	0	0	0	0	0	0	0	0	0
1469	0	0	0	0	0	1	0	2	2
1470	0	0	0	0	0	0	0	0	0
1471	0	0	0	0	0	0	0	0	0
1472	0	0	0	0	0	0	0	0	0
1473	0	0	0	0	0	0	0	0	0
1474	0	0	0	0	0	0	0	0	0
1475	0	0	0	0	0	0	0	0	0
1476	0	0	0	0	0	0	0	0	0
1477	0	0	0	0	0	0	0	0	0
1478	0	0	0	0	0	0	0	0	0
1479	0	0	0	0	0	0	0	0	0
1480	0	0	0	0	0	0	0	0	0
1481	0	0	0	0	0	0	0	0	0
1482	0	0	0	0	0	0	0	0	0

1483	0	0	0	0	0	0	0	0	0
1484	0	0	0	0	0	0	0	0	0
1485	0	0	0	0	0	0	0	0	0
1486	0	0	0	0	0	0	0	0	0
1487	0	0	0	0	0	0	0	0	0
1488	0	0	0	0	0	0	0	0	0
1489	0	0	0	0	0	0	0	0	0
1490	0	0	0	0	0	0	0	0	0
1491	0	0	0	0	0	0	0	0	0
1492	0	0	0	0	0	0	0	0	0
1493	0	0	0	0	0	0	0	0	0
1494	0	0	0	0	0	0	0	0	0
1495	0	0	0	0	0	0	0	0	0
1496	0	0	0	0	0	0	0	0	0
1497	0	0	0	0	0	0	0	0	0
1498	0	0	0	0	0	0	0	0	0
1499	0	0	0	0	0	0	0	0	0
1500	0	0	0	0	0	0	0	0	0
1501	0	0	0	0	0	0	0	0	0
1502	0	0	0	0	0	0	0	0	0
1503	0	0	0	0	0	0	0	0	0
1504	0	0	0	0	0	0	0	0	0
1505	0	0	0	0	0	0	0	0	0
1506	0	0	0	0	0	0	0	0	0
1507	0	0	0	0	0	0	0	0	0
1508	0	0	0	0	0	0	0	0	0
1509	0	0	0	0	0	0	0	0	0
1510	0	0	0	0	0	0	0	0	0
1511	0	0	0	0	0	0	0	0	0
1512	0	0	0	0	0	0	0	0	0
1513	0	0	0	0	0	0	0	0	0
1514	0	0	0	0	0	0	0	0	0
1515	0	0	0	0	0	0	0	0	0
1516	0	0	0	0	0	0	0	0	0
1517	0	0	0	0	0	0	0	0	0
1518	0	0	0	0	0	0	0	0	0
1519	0	0	0	0	0	0	0	0	0
1520	0	0	0	0	0	0	0	0	0
1521	0	0	0	0	0	0	0	0	0
1522	0	0	0	0	0	0	0	0	0
1523	0	0	0	0	0	0	0	0	0
1524	0	0	0	0	0	0	0	0	0
1525	0	0	0	0	0	0	0	0	0
1526	0	0	0	0	0	0	0	0	0
1527	0	0	0	0	0	0	0	0	0
1528	0	0	0	0	0	0	0	0	0
1529	0	0	0	0	0	0	0	0	0
1530	0	0	0	0	0	0	0	0	0
1531	0	0	0	0	0	0	0	0	0
1532	0	0	0	0	0	0	0	0	0
1533	0	0	0	0	0	0	0	0	0
1534	0	0	0	0	0	0	0	0	0
1535	0	0	0	0	0	0	0	0	0

1536	0	0	0	0	0	0	0	0	0
1537	0	0	0	0	0	0	0	0	0
1538	0	0	0	0	0	0	0	0	0
1539	0	0	0	0	0	0	0	0	0
1540	0	0	0	0	0	0	0	0	0
1541	0	0	0	0	0	0	0	0	0
1542	0	0	0	0	0	0	0	0	0
1543	0	0	0	0	0	0	0	0	0
1544	0	0	0	0	0	0	0	0	0
1545	0	0	0	0	0	0	0	0	0
1546	0	0	0	0	0	0	0	0	0
1547	0	0	0	0	0	0	0	0	0
1548	0	0	0	0	0	0	0	0	0
1549	0	0	0	0	0	0	0	0	0
1550	0	0	0	0	0	0	0	0	0
1551	0	0	0	0	0	0	0	0	0
1552	0	0	0	0	0	0	0	0	0
1553	0	0	0	0	0	0	0	0	0
1554	0	0	0	0	0	0	0	0	0
1555	0	0	0	0	0	0	0	0	0
1556	0	0	0	0	0	0	0	0	0
1557	0	0	0	0	0	0	0	0	0
1558	0	0	0	0	0	0	0	0	0
1559	0	0	0	0	0	0	0	0	0
1560	0	0	0	0	0	0	0	0	0
1561	0	0	0	0	0	0	0	0	0
1562	0	0	0	0	0	0	0	0	0
1563	0	0	0	0	0	0	0	0	0
1564	0	0	0	0	0	0	0	0	0
1565	0	0	0	0	0	0	0	0	0
1566	0	0	0	0	0	0	0	0	0
1567	0	0	0	0	0	0	0	0	0
1568	0	0	0	0	0	0	0	0	0
1569	0	0	0	0	0	0	0	0	0
1570	0	0	0	0	0	0	0	0	0
1571	0	0	0	0	0	0	0	0	0
1572	0	0	0	0	0	0	0	0	0
1573	0	0	0	0	0	0	0	0	0
1574	0	0	0	0	0	0	0	0	0
1575	0	0	0	0	0	0	0	0	0
1576	0	0	0	0	0	0	0	0	0
1577	0	0	0	0	0	0	0	0	0
1578	0	0	0	0	0	0	0	0	0
1579	0	0	0	0	0	0	0	0	0
1580	0	0	0	0	0	0	0	0	0
1581	0	0	0	0	0	0	0	0	0
1582	0	0	0	0	0	0	0	0	0
1583	0	0	0	0	0	0	0	0	0
1584	0	0	0	0	0	0	0	0	0
1585	0	0	0	0	0	0	0	0	0
1586	0	0	0	0	0	0	0	0	0
1587	0	0	0	0	0	0	0	0	0
1588	0	0	0	0	0	0	0	0	0

1589	0	0	0	0	0	0	0	0	0
1590	0	0	0	0	0	0	0	0	0
1591	0	0	0	0	0	0	0	0	0
1592	0	0	0	0	0	0	0	0	0
1593	0	0	0	0	0	0	0	0	0
1594	0	0	0	0	0	0	0	0	0
1595	0	0	0	0	0	0	0	0	0
1596	0	0	0	0	0	0	0	0	0
1597	0	0	0	0	0	0	0	0	0
1598	0	0	0	0	0	0	0	0	0
1599	0	0	0	0	0	0	0	0	0
1600	0	0	0	0	0	0	0	0	0
1601	0	0	0	0	0	0	0	0	0
1602	0	0	0	0	0	0	0	0	0
1603	0	0	0	0	0	0	0	0	0
1604	0	0	0	0	0	0	0	1	1
