# Supplementary material for: Unraveling the Design Principle for Motif Organization in Signaling Networks
Source: PLoS One. 2011 Dec 2;6(12):e28606. doi: 10.1371/journal.pone.0028606 (PMC3228783; doi:10.1371/journal.pone.0028606)
Supplement: Table S3 — Functions assigned to the genes in the signaling network. Function of all the nodes present in the human signaling network is listed in this table. This data was directly used from an already published manuscript (30). (PDF) [file pone.0028606.s004.pdf]

Supplementary Table S3

Node Number	Gene Name	Function
1	14-03-2003	A-A
2	4-1BB	Receptor
3	4-1BBL	Cytokine
4	A20	GF
5	a2-Antiplasmin	NA
6	Ab-R	Receptor
7	ABI2	SP
8	ABL1	TF
9	AC	Generics
10	ACC	Kinase
11	ACHRE	Receptor
12	ACT	SP
13	ACTG	Cytoskeleton
14	ACTN1	Generics
15	ACTR	Generics
16	ADAM12	Caspases
17	AFX	TF
18	AGTR1	Transmenmbrane Proteins
19	AGTR2	Receptor
20	AHR	TF
21	AKAP13	A-Kinase Anchor Proteins
22	AKT	Kinase
23	ALDOA	Generics
24	ALK3	Receptor
25	AMP	Molecule
26	AMPK	Kinase
27	ANF	Generics
28	APAF1	P-A
29	APC	SP
30	APE1	SP
31	APLP1	SP
32	APO2L	Ligand
33	APO3L	Ligand
34	APOER2	Receptor
35	ARF1	Molecule
36	ARHGAP1	GAP
37	ARHGAP4	GAP
38	ARHGAP5	GAP
39	ARHGAP6	GAP
40	ARHGEF1	GEF
41	ARHGEF11	GEF
42	ARHGEF5	GEF
43	ARNT	TF
44	ARP2	Generics
45	ARP3	Generics
46	ASK1	Kinase
47	ATF2	TF
48	ATM	Kinase
49	ATP	Molecule
50	ATR	Kinase
51	ATRIP	Generics
52	AVP	SP

53	AXIN	SP
54	B2AR	MDTP
55	BAD	P-A
56	BAK	P-A
57	BAX	P-A
58	BCL2	A-A
59	BCL2L1	A-A
60	BID	P-A
61	BIK	SP
62	BIRC5	Generics
63	BLNK	Adapter
64	BMAL1	TF
65	BMPR2	Receptor
66	BRCA1	TF
67	BRCA2	Generics
68	BTK	Kinase
69	BTRC	Adapter
70	C3G	Generics
71	Ca++	Ion
72	CABIN1	SP
73	CAD	TF
74	PPP3R2	Phosphatase
75	Calpain1	Caspases
76	Calpain2	Caspases
77	Calpastatin	inhibitor
78	CAM	SP
79	CAMK1	Kinase
80	CAMK2	Kinase
81	CAMP	Molecule
82	CAP	SP
83	CARM1	SP
84	CASP1	Caspases
85	CASP10	Caspases
86	CASP2	Caspases
87	CASP3	Caspases
88	CASP6	Caspases
89	CASP7	Caspases
90	CASP8	Caspases
91	CASP9	Caspases
92	Catalase	SP
93	CBL	Generics
94	CBP	Kinase
95	CCL5	Chemokines
96	CCNA2	Generics
97	CCNB1	Generics
98	CCND1	Generics
99	CCND2	Generics
100	CCND3	Generics
101	CCNE1	Generics
102	CCR3	MDTP
103	CCR5	Multiple Domain Trans-membrane Proteins
104	CCT4	Generics
105	Cd++	NA
106	CD14	Kinase

107	CD36	Receptor
108	CD4	Receptor
109	CD40	MDTP
110	CD40L	MDTP
111	CD45	Phosphatase
112	CDC25	Phosphatase
113	CDC25A	Phosphatase
114	CDC25C	Phosphatase
115	CDC34	Generics
116	CDC42	Kinase
117	CDH1	Generics
118	CDK1	Kinase
119	CDK2	Kinase
120	CDK4	Kinase
121	CDK5	Kinase
122	CDK6	Kinase
123	CDK7	Kinase
124	CEBPA	TF
125	CEM15	SP
126	CHK1	Kinase
127	CHK2	Kinase
128	CHN1	GAP
129	CHORDIN	SP
130	CHREBP	TF
131	cIAP	A-A
132	cIAP1	A-A
133	c-JUN	TF
134	CK1a	Kinase
135	CK1d	Kinase
136	CK1e	Kinase
137	CK2	Kinase
138	CKS1A	Generics
139	CLOCK	TF
140	cMAF	TF
141	c-Myc	TF
142	COFILIN2	Generics
143	CPI	NA
144	CREB	TF
145	CREM	TF
146	CRK	Generics
147	CRKL	Kinase
148	CRY	SP
149	CSF1R	Receptor
150	CSK	Kinase
151	CSX	TF
152	CTBP1	Adapter
153	CTIP	Adapter
154	CTNNB1	Generics
155	CTSD	Generics
156	CUL1	Generics
157	CXCR4	Multiple Domain Trans-membrane Proteins
158	CYCS	Generics
159	CYP19A1	NA
160	D1	Multiple Domain Trans-membrane Proteins

161	D2	Multiple Domain Trans-membrane Proteins
162	D4-GDI	Generics
163	DAB1	SP
164	DAG	Adapter
165	DARPP-32	Generics
166	DAXX	P-A
167	DELTA	Generics
168	DG	Adapter
169	DHPG	NA
170	DIA	Kinase
171	DKK	Signal transducer
172	DNAPK	Kinase
173	DOCK180	Adapter
174	Dopamine	NA
175	DP1	TF
176	DP103	TF
177	DR3	Receptor
178	DR4	Receptor
179	DR5	Receptor
180	DREAM	TF
181	DSH	SP
182	dsRNA	RNA
183	Dynein	SP
184	E12	NA
185	E1A	TF
186	E2	Generics
187	E2F1	TF
188	E2F4	TF
189	E3	Ligases
190	EBP	SP
191	EBS	Adapter
192	ECM	NA
193	ECSIT	Generics
194	EDG1	Multiple Domain Trans-membrane Proteins
195	EDNRA	Multiple Domain Trans-membrane Proteins
196	EGF	GF
197	EGFR	Receptor
198	EGR1	TF
199	EGR2	TF
200	EGR3	TF
201	EIF-4EBP	SP
202	EIF2A	TF
203	EIF2B	TF
204	EIF2G	TF
205	EIF3	SP
206	EIF4A	SP
207	EIF4B	SP
208	EIF4E	SP
209	EIF4F	SP
210	EIF5	SP
211	ELK1	TF
212	EPAC1	NA
213	EPO	GF
214	EPOR	Receptor

215 ER	TF
216 Era	Receptor
217 ERBB2	Receptor
218 ERE	NA
219 ERK1	Kinase
220 ERK2	Kinase
221 ERK5	Kinase
222 Estrogen	Molecule
223 ETS1	TF
224 ETS2	TF
225 F10	Caspases
226 F11	Caspases
227 F13	GF
228 F2	Caspases
229 F3	GF
230 F7	Caspases
231 F9	Caspases
232 FADD	P-A
233 FAK	Kinase
234 FAN	Generics
235 FANCA	SP
236 FANCC	SP
237 FANCD2	SP
238 FANCE	SP
239 FANCF	SP
240 FANCG	SP
241 FAP	Phosphatase
242 FAS	Receptor
243 FASLG	MDTP
244 FBI1	TF
245 FBW7	Generics
246 FBXO6	SP
247 Fe	NA
248 Fibrinogen	NA
249 FKHRL1	TF
250 FLT3	Generics
251 FODRIN	SP
252 Forskolin	NA
253 FOS	TF
254 FOSB	TF
255 FRAT1	SP
256 FRIZZLED	Receptor
257 FSH	GF
258 FSHR	Multiple Domain Trans-membrane Proteins
259 FXR	SP
260 FYN	Kinase
261 G1	NA
262 G2	NA
263 G3PD	Generics
264 GA	SP
265 GAA	NA
266 GAB1	SP
267 GADD45	Adapter
268 Gai	SP

269	GAP	SP
270	GAQ	SP
271	GAS	TF
272	GAS2	NA
273	GATA3	TF
274	GATA4	TF
275	Gb	SP
276	GC1	SP
277	GC2	SP
278	GCAP	SP
279	GCK	Kinase
280	GCN2	Kinase
281	GCSF	GF
282	GDP	SP
283	GEF	Rac GDP-GTP exchange factors
284	GELSOLIN	Generics
285	GFAP	SP
286	GFR	Receptor
287	GH	GF
288	GHR	Receptor
289	Gia	SP
290	GLI1	SP
291	GLI2	SP
292	GLI3	SP
293	glucocorticoid	Molecule
294	GLUT4	Adapter
295	GMCSF	GF
296	GNA12	SP
297	GNA13	SP
298	GNA16	SP
299	GPCR	Multiple Domain Trans-membrane Proteins
300	GPx	Generics
301	GR	TF
302	GRB2	SP
303	GRE	Generics
304	GREB1	Generics
305	GROUCHO	Adapter
306	GSH	Generics
307	GSK3A	Kinase
308	GSK3B	Kinase
309	GSR	Generics
310	GSSG	Generics
311	GTP	SP
312	Gy	SP
313	GZMB	SP
314	H1	DNA Binders
315	H2O2	Molecule
316	HAND1	TF
317	HAND2	TF
318	HBX	TF
319	HDAC	Deacetylase
320	HDAC1	Deacetylase
321	HDAC2	Deacetylase
322	HDAC3	Deacetylase

323	HDAC5	Phosphatase
324	HGF	GF
325	HIF1A	TF
326	HMG1	Generics
327	HMG2	Generics
328	HMGN1	Generics
329	HO	Molecule
330	HOP	Adapter
331	HRI	Kinase
332	HSP27	Generics
333	Hsp40	Generics
334	hsp70	SP
335	Hsp90	Generics
336	HSPC	SP
337	HUR	TF
338	HUS1	Generics
339	Hypoxia	Molecule
340	IAP	SP
341	IC261	inhibitor
342	ICAD	GF
343	I-FLICE	A-A
344	IFNy	Cytokine
345	IFN-yR	Receptor
346	IGF1	GF
347	IGF1R	Receptor
348	IKBA	TF
349	IKBB	SP
350	IKK2	Kinase
351	IKKA	Kinase
352	IKKy	Kinase
353	IL11	GF
354	IL12RB2	Receptor
355	IL1A	GF
356	IL1B	GF
357	IL1R	Receptor
358	IL2	GF
359	IL3	GF
360	IL4	GF
361	IL6	GF
362	IL8	Cytokine
363	IL9	Cytokine
364	Importin	Generics
365	INSR	Receptor
366	Insulin	Molecule
367	CALM3	Ion-binding
368	IP3R	Receptor
369	IRAK	Kinase
370	IRS1	Adapter
371	IRSP53	SP
372	ISRE	Adapter
373	ITGA1	Generics
374	ITGB1	Generics
375	ITGB3	Generics
376	JAK1	Kinase

377	JAK2	Kinase
378	JNK	Kinase
379	JNKK1	Kinase
380	JUNB	TF
381	JUND	TF
382	K+	NA
383	KB	Kinase
384	KIF17B	Generics
385	LAMA1	SP
386	LAMB1	SP
387	LAMC1	SP
388	LAT	SP
389	LCK	Kinase
390	LEP	Cytokine
391	LEPR	Receptor
392	LIMK1	Kinase
393	LMNA	SP
394	LMNB1	SP
395	LMNB2	SP
396	LPL	Receptor
397	LPS	TF
398	LRP6	SP
399	LXR	SP
400	LYN	Kinase
401	MADD	SP
402	MAL	TF
403	MAP4K1	Kinase
404	MAP4K5	Kinase
405	MAPK11	Kinase
406	MAPK13	Kinase
407	MAPK9	TF
408	MAPKAP2	Kinase
409	MARCKS	Generics
410	MBD3	TF
411	MD-2	SP
412	mDIA	Kinase
413	MDM2	Adapter
414	MEF2	TF
415	MEF2C	TF
416	MEF2D	TF
417	MEK1	Kinase
418	MEK2	Kinase
419	MEK3	Kinase
420	MEK6	Kinase
421	MEKK	Kinase
422	MET	TF
423	METS	TF
424	MGLUR1	Multiple Domain Trans-membrane Proteins
425	MIG1	Generics
426	MIP1B	Ligand
427	MITF	TF
428	MITR	Deacetylase
429	MKK7	Kinase
430	MKP1	Phosphatase

431	MLC	Kinase
432	MLCK	Kinase
433	MLCP	Phosphatase
434	MLK1	Kinase
435	MNK1	Kinase
436	MPR	MDTP
437	MRE11	Transferase
438	MRLC	SP
439	MSK1	Kinase
440	MTOR	Kinase
441	MYD88	SP
442	myosin	SP
443	MYT1	TF
444	NAP	SP
445	NBS1	TF
446	NEDD8	SP
447	NFAT	TF
448	NFkB	TF
449	NFYA	TF
450	NFYB	TF
451	NFYC	TF
452	NGF	GF
453	NGFR	Receptor
454	NIK	Kinase
455	NLK	Kinase
456	NME1	SP
457	NOS1	Phospholipase
458	NOS3	Phospholipase
459	NOX1	Kinase
460	nPKC	Kinase
461	NTF5	GF
462	NTH1	Kinase
463	NTRK1	Receptor
464	NUR77	Gene
465	O2	Molecule
466	p107	TF
467	p120	SP
468	P13K	Kinase
469	P130	TF
470	P130Cas	Adapter
471	p14ARF	CellCycle
472	P15	CellCycle
473	p16	SP
474	p190RhoGAP	SP
475	P2	Generics
476	P21	SP
477	p25	Generics
478	p27	CellCycle
479	p300	TF
480	p34	SP
481	P35	Generics
482	p38	Kinase
483	P50	TF
484	p53	TF

485	p6	SP
486	p65	TF
487	p67phox	SP
488	p70s6K	Kinase
489	P73	TF
490	p90RSK	Kinase
491	PABP	RNA binding
492	PAI1	Generics
493	PAK1	Kinase
494	PAK2	Kinase
495	PAR1	Receptor
496	PAR4	Receptor
497	PARP	Transferase
498	PBP	TF
499	PCLy	Kinase
500	PCNA	TF
501	PDE2	NA
502	PDE6A	SP
503	PDE6B	SP
504	PDE6G	SP
505	PDGFB	Generics
506	PDGFRA	Receptor
507	PDK1	Kinase
508	PDK2	Kinase
509	PDZK1	Generics
510	PELP1	SP
511	PER	Generics
512	PERK	Kinase
513	PFN1	Generics
514	PGC1A	TF
515	PI3K	Kinase
516	PI3Ky	Kinase
517	PI-4-P5K	Kinase
518	PIB5PA	hydrolase
519	INPP5A	hydrolase
520	PIR	SP
521	PKAc	Kinase
522	PKC	Kinase
523	PKCA	Kinase
524	PKCE	Kinase
525	PKLR	Kinase
526	PKN	Kinase
527	PKR	Kinase
528	PLA2	Phospholipase
529	PLASMIN	Caspases
530	PLCb	Phospholipase
531	PLCD1	Phosphatase
532	PLCE	Phospholipase
533	PLCy	Receptor
534	PLD1	Phospholipase
535	PLG	Generics
536	PLK1	Kinase
537	PLK3	Kinase
538	POLR1B	Generics

539	POR1	SP
540	PP1	Phosphatase
541	PP2A	Phosphatase
542	PP2B	Phosphatase
543	PP32	SP
544	PPARA	Receptor
545	PPARB	Receptor
546	PPARG	Receptor
547	PRAK	Kinase
548	PRF1	NA
549	PSEN1	Receptor
550	PTC	Multiple Domain Trans-membrane Proteins
551	PTEN	Phosphatase
552	PTK	Kinase
553	PTPB1	Kinase
554	PXN	Generics
555	RAD1	Generics
556	RAD17	Generics
557	RAD50	P-A
558	RAD51	DNA Binders
559	RAD9	Generics
560	RAF1	Kinase
561	RAIDD	P-A
562	RAL	Kinase
563	RALBP1	Kinase
564	RALGDS	Guanine Exchange Factor
565	RAN	Kinase
566	RANBP1	SP
567	RANBP2	SP
568	RanGAP	Transferase
569	RAP1A	Kinase
570	RAP1B	Kinase
571	RAR	TF
572	RAC1	Receptor
573	RASGRF	Chemokines
574	RB	TF
575	RELA	TF
576	RELN	Extracellular Protein
577	REQ	TF
578	Rho	SP
579	RHOA	Kinase
580	RhoGDP	Molecule
581	RhoGTP	Molecule
582	RHOGEFs	exchange factors
583	RIIa	SP
584	RIIb	SP
585	RIP	Kinase
586	ROC1	Generics
587	ROCK1	Kinase
588	ROCK2	Kinase
589	ROS	Kinase
590	RPS6	SP
591	RSK2	Kinase
592	RXR	TF

593	S1P	SP
594	PSMC4	SP
595	SAC	NA
596	SAG	Generics
597	Sara	SP
598	SCF	stem cell factor
599	SDF1	Adapter
600	SERPINC1	Generics
601	SET	SP
602	SHC	Adapter
603	SHP1	Phosphatase
604	SHP2	Phosphatase
605	Sin3	Generics
606	SKIP	NA
607	SKP1	Generics
608	SKP2	TF
609	SMAD1	TF
610	SMAD2	TF
611	SMAD3	TF
612	SMAD4	TF
613	SMAD5	TF
614	SMAD6	TF
615	SMAD7	TF
616	SMO	Multiple Domain Trans-membrane Proteins
617	SMPD1	Generics
618	SMPD2	Generics
619	SMRT	TF
620	SNAI1	TF
621	SNCA	SP
622	SnoN	Adapter
623	SOD	Generics
624	SODD	A-A
625	SOS1	Phospholipase
626	SOS2	A-A
627	SP1	TF
628	SP3	TF
629	SPHK1	Kinase
630	SPRY	Generics
631	SPTAN1	SP
632	SRC	Kinase
633	SRF	TF
634	STAT1	TF
635	STAT2	TF
636	STAT3	TF
637	STAT4	TF
638	STAT5	TF
639	SUFU	SP
640	SUMO1	Small Ubiquitin-Like Modifier
641	SYK	Kinase
642	Ta	Generics
643	TAB1	Kinase
644	TAB2	Kinase
645	TAK1	Kinase
646	TALIN	Generics

647	TANK	SP
648	TAU	SP
649	TAX	NA
650	Tb	Generics
651	TBX2	TF
652	TCF	TF
653	TERT	DNA Binders
654	TFF1	Trefoil Factors
655	TFF2	Trefoil Factors
656	TGFB	GF
657	TGFBR1	Receptor
658	TGFBR2	Receptor
659	TGFBR3	Receptor
660	TID1	SP
661	TIF2	Generics
662	TIP1	Receptor
663	TIRAP	Generics
664	TLR2	Receptor
665	TLR3	Receptor
666	TLR4	Receptor
667	TLR6	Receptor
668	TLR7	Receptor
669	TLR9	Receptor
670	TNF	Tumor Necrosis Factors
671	TNFB	Cytokine
672	TNFR1	Receptor
673	TNFR2	Receptor
674	TNKS	NA
675	TOB	TF
676	TOLLIP	Generics
677	TopoII	Generics
678	TPA	SP
679	TRADD	SP
680	TRAF1	SP
681	TRAF2	SP
682	TRAF3	SP
683	TRAF6	Generics
684	TREH	NA
685	TRIO	GEF
686	TRX	NA
687	TSA	Trichostatin
688	TSC1	SP
689	TSC2	SP
690	TSP1	Generics
691	TUBBY	TF
692	TWIST	TF
693	Ty	Generics
694	TYK2	Kinase
695	Ub	SP
696	UBE2M	Generics
697	VAV	SP
698	VAV2	SP
699	VCL	SP
700	VEGF	Generics

701	VEGFR	Receptor
702	VELI1	NA
703	VIL2	Generics
704	VLDLR	Receptor
705	VPAC2R	Multiple Domain Trans-membrane Proteins
706	WAVE1	SP
707	WAVE2	SP
708	WAVE3	SP
709	WIF1	SP
710	WNT	SP
711	XAP2	Molecule
712	XDH	Generics
713	XIAP	Generics
714	ZAP70	Kinase
715	ZEB1	TF
716	A1R	Receptor
717	A2AR	Receptor
718	AA	Lipid
719	ABP1	Adapter
720	AC1	Cyclase
721	AC2	Cyclase
722	AC5	Cyclase
723	ACH	Receptor
724	ACTIN	Cytoskeleton
725	ADAPTIN	Adapter
726	ADDUCIN	Adapter
727	ADENOSINE	Ligand
728	ADPRIBSYCYCLASE	Synthase
729	AKAP	Adapter
730	AKAP15	Adapter
731	ALPHA1AR	Receptor
732	ALPHA2AR	Receptor
733	ALPHA7NACHR	Receptor
734	ALPHAACTININ	Cytoskeleton
735	ALPHACATENIN	Cytoskeleton
736	AMISYN	Vesicle
737	AMPAR	Channel
738	AMPHIPHYSIN	Adapter
739	ANANDAMIDE	Ligand
740	ANKYRIN	Cytoskeleton
741	AP2	Adapter
742	ANAPC1	Adapter
743	APOLLON	Ubiquitinase
744	APS	Adapter
745	ARF	GTPase
746	ARFGAP	GAP
747	ARFGEF	GEF
748	ARIP2	Adapter
749	ARNO	GEF
750	ARP23	Cytoskeleton
751	ATF1	TF
752	BAG1	Adapter
753	BAP31	Adapter
754	bARK	Kinase

755	BDNF	Ligand
756	BETAARRESTIN	Adapter
757	BIM	Bcl2Family
758	BIP	Chaperone
759	BR1R	Receptor
760	BRADYKININ	Ligand
761	bRAF	Kinase
762	DFFB	DNase
763	cADPR	Messenger
764	CALBRAIN	Inhibitor
765	CALCINEURIN	Phosphatase
766	CALMODULIN	Activator
767	CALNEXIN	Chaperone
768	CALRETICULIN	Chaperone
769	CAMK2A	Kinase
770	CAMKIV	Kinase
771	CAMKK	Kinase
772	CAMKPASE	Phosphatase
773	cAMPGEFII	GEF
774	CAPRI	GAP
775	CASK	Kinase
776	CASKIN	Vesicle
777	CAVEOLIN	Adapter
778	CB1R	Receptor
779	CB2R	Receptor
780	CBLB	Ubiquitinase
781	CEK1	Kinase
782	CERAMIDE	Lipid
783	CGMP	Messenger
784	CHAPSYN110	Adapter
785	CHLORIDE	Ion
786	CHOLESTEROL	Lipid
787	cIAP2	Inhibitor
788	CIPP	Adapter
789	CITRON	Kinase
790	CLATHRIN	Vesicle
791	CLIP170	Adapter
792	COFILIN1	Cytoskeleton
793	COLLAGEN	Matrix
794	COMPLEXIN	Vesicle
795	CORTACTIN	Cytoskeleton
796	CPI17	Inhibitor
797	CRE	DNA Sequences
798	CRIPT	Adapter
799	CRMP2	Activator
800	CSP	Adapter
801	CYTOCHROMEC	DualRole
802	D1R	Receptor
803	D2R	Receptor
804	D3R	Receptor
805	DAP	Adapter
806	DAPK	Kinase
807	dATP	Messenger
808	DCP1	Ribosome

809	DENSIN	Adapter
810	DGK	Kinase
811	DISHEVELED	Adapter
812	DLK	Kinase
813	DNAJ	Chaperone
814	DOC2	Vesicle
815	DOK	Adapter
816	DOPR	Receptor
817	DRE	DNA Binders
818	DYNAMIN	GTPase
819	DYNEIN	SP
820	DYRK	Kinase
821	EEF1A2	Elongation
822	EEF2	GTPase
823	EEF2K	GTPase
824	EFA6	GEF
825	EIF1A	Ribosome
826	EIF3S1	Ribosome
827	EIF4	Ribosome
828	EIF4G	Ribosome
829	ENDOPHILIN	Vesicle
830	ENKEPHALIN	Ligand
831	EPHB2	Receptor
832	EPHRIN	Ligand
833	ERBIN	Adapter
834	FASCIN	CellAdhesion
835	FER	Kinase
836	FIBRONECTIN	Matrix
837	FILAMIN	Cytoskeleton
838	FIVEHT1AR	Receptor
839	FIVEHT1CR	Receptor
840	FIVEHT2AR	Receptor
841	FIVEHT4R	Receptor
842	FKBP	Adapter
843	FORKHEAD	TF
844	FOUREBP1	TF
845	FRAT2	Adapter
846	GABA	Ligand
847	GABAAR	Receptor
848	GABABR	Receptor
849	GABARAP	Adapter
850	GAIP	GAP
851	GALPHAI	Gprotein
852	GALPHAO	Gprotein
853	GALPHAS	Gprotein
854	GALPHAZ	Gprotein
855	GAP43	Adapter
856	GAT1	Transporter
857	GBETAGAMMA	Gprotein
858	GDNF	Ligand
859	GEPHYRIN	Adapter
860	GIRK	Channel
861	GIT1	Adapter
862	GKAP	Adapter

863	GLUTAMATE	Ligand
864	GLYCINE	Ligand
865	GLYR	Receptor
866	GLYT1	Transporter
867	GRB10	Adapter
868	GRIP	Adapter
869	GRIT	GAP
870	GRP94	Chaperone
871	HCK	Kinase
872	HIPPOCALCIN	Adapter
873	HOMER	Adapter
874	I1	Inhibitor
875	ICA512	Receptor
876	ILK	Kinase
877	INHIBITOR2	Inhibitor
878	IP3	Lipid
879	IP6K2	Kinase
880	IQGAP	GAP
881	IRS2	Adapter
882	JIP	Adapter
883	KAR	Channel
884	KINESIN	Cytoskeleton
885	KIR21	Channel
886	KIR23	Channel
887	KIR41	Channel
888	KOPR	Receptor
889	KSR	Kinase
890	KV11	Channel
891	KV12	Channel
892	KV14	Channel
893	KV41	Channel
894	KV42	Channel
895	L1	CellAdhesion
896	LARG	GEF
897	LEF	TF
898	LEF1	TF
899	LRP	Receptor
900	LTYPECA	Channel
901	M1R	Receptor
902	M2R	Receptor
903	M4R	Receptor
904	MALS	Adapter
905	MAP1A	Cytoskeleton
906	MAP1B	Cytoskeleton
907	MAP2	Cytoskeleton
908	MBP	Cytoskeleton
909	MEF2B	TF
910	MAP3K2	Kinase
911	MAP4K3	Kinase
912	MAP4K6	Kinase
913	MEKK2	Kinase
914	MEKK3	Kinase
915	MEKK4	Kinase
916	MERLIN	Adapter

917	MGLUR7	Receptor
918	MINT	Vesicle
919	MKP5	Phosphatase
920	MLK	Kinase
921	MLK3	Kinase
922	CARD14	Kinase
923	MOPR	Receptor
924	MOR	Receptor
925	MP1	Adapter
926	mRNA	RNA
927	MST3	Adapter
928	MUNC13	Vesicle
929	MUNC18	Vesicle
930	MUSK	Receptor
931	MYOSIN	SP
932	MYOSINV	Cytoskeleton
933	N41	Adapter
934	NAIP	Inhibitor
935	NAKED	Antagonist
936	NASCENTCHAIN	Peptide
937	NCADHERIN	CellAdhesion
938	NCK	Adapter
939	NCS1	Adapter
940	NE	Ligand
941	NEURABIN	Adapter
942	NEUREXIN	CellAdhesion
943	NEUROFIBROMIN	GAP
944	NEUROLIGIN	CellAdhesion
945	NFH	Cytoskeleton
946	NFM	Cytoskeleton
947	NHERF	Adapter
948	NMDAR	Receptor
949	NO	Molecule
950	NOPR	Receptor
951	NRCAM	CellAdhesion
952	NRG	Ligand
953	NTYPECA	Channel
954	P1433	Adapter
955	P35611	Activator
956	TP53BP1	TF
957	PA	Messenger
958	PAF	Ligand
959	PAFR	Receptor
960	PAR2	Receptor
961	PCAF	TF
962	PDE1A	PDE
963	PDE1B	PDE
964	PDE1C	PDE
965	PDE3A	PDE
966	PDE3B	PDE
967	PDE4A	PDE
968	PDE4B	PDE
969	PDE4C	PDE
970	PDE4D	PDE

971	PDE5A	PDE
972	PDGF	Ligand
973	PDGFR	Receptor
974	PDI	Chaperone
975	PDPK1	Kinase
976	PDZGEF	GEF
977	pGC	Cyclase
978	PICCOLO	Adapter
979	PICK1	Adapter
980	PIN1	TF
981	PIP2	Receptor
982	PIP3	Lipid
983	PIP5K	Kinase
984	PKA	Kinase
985	PKD	Kinase
986	PKG	Kinase
987	PKI	Inhibitor
988	PROKR1	Kinase
989	PLA2P	Phospholipase
990	PLD	Adapter
991	PMCA	Pump
992	POTASSIUM	Ion
993	PPP1CC	Phosphatase
994	PPP2R5C	Phosphatase
995	PP2C	Phosphatase
996	PP5	Phosphatase
997	PQCaCh	Channel
998	PRK2	Kinase
999	PRMT1	Methylase
1000	PROFILIN	Cytoskeleton
1001	PSD93	Adapter
1002	PSD95	Adapter
1003	PTP1B	Phosphatase
1004	PTP1D	Phosphatase
1005	PTPA	Phosphatase
1006	RAB3	GTPase
1007	RABGDI	Inhibitor
1008	RABPHILIN	Vesicle
1009	RACK	Adapter
1010	RADIXIN	Adapter
1011	RAP1	GTPase
1012	RAP1GAP	GAP
1013	RAP2	GTPase
1014	RAS	GTPase
1015	RASGAP	GAP
1016	RASGRP1	GAP
1017	RASGRP3	GEF
1018	REPS1	Adapter
1019	RET	Receptor
1020	RGS2	Gprotein
1021	RGS4	GAP
1022	RHEB	Adapter
1023	RIC	Adapter
1024	RICS	Adapter

1025	RIM	Adapter
1026	RIN	GTPase
1027	RIN1	Activator
1028	RSK	Kinase
1029	RTYPECA	Channel
1030	RYR	Channel
1031	S6	Adapter
1032	S60	Ribosome
1033	SAM68	Adapter
1034	SAP102	Adapter
1035	SAP97	Adapter
1036	SAPAP	Adapter
1037	SEC61P	Channel
1038	SEC63P	Adapter
1039	SEK	Kinase
1040	SEROTONIN	Ligand
1041	SHANK	Adapter
1042	SH3KBP1	Phosphatase
1043	SIAH	Adapter
1044	SIVA1	Adapter
1045	SNAP25	Vesicle
1046	SNAPIN	Vesicle
1047	SODIUM	Ion
1048	SORCIN	Adapter
1049	SPAL	GAP
1050	SPECTRIN	Cytoskeleton
1051	SPINOPHILIN	Cytoskeleton
1052	SRE	DNA Binders
1053	SRP54	Adapter
1054	SRPR	Receptor
1055	SS	Ligand
1056	SSCAM	Adapter
1057	SSTR1	Receptor
1058	SSTR2	Receptor
1059	STARGAZIN	Chaperone
1060	STEP	Phosphatase
1061	SV2A	Vesicle
1062	SYNAPSIN	Vesicle
1063	SYNAPTOBREVIN	Vesicle
1064	SYNAPTOPHYSIN	Vesicle
1065	SYNAPTOTAGMIN	Vesicle
1066	SYNDECAN	Receptor
1067	SYNGAP	GEF
1068	SYNTAPHILIN	Vesicle
1069	SYNTAXIN	Vesicle
1070	SYNTENIN	Adapter
1071	SYNTROPHIN	Adapter
1072	TAMALIN	Adapter
1073	TBR1	TF
1074	TCF7	TF
1075	TFIIB	TF
1076	TFIID	TF
1077	THC	Ligand
1078	TIAM1	GEF

1079	TRE	DNA Binders
1080	TRKB	Receptor
1081	tRNA	RNA
1082	TRYPSIN	Protease
1083	TUBULIN	Cytoskeleton
1084	VAMP	Vesicle
1085	VASP	Adapter
1086	VDAC2	Channel
1087	VILIP	Adapter
1088	VITRONECTIN	Matrix
1089	VRK1	Kinase
1090	WNT6	Ligand
1091	YES	Kinase
1092	YOTIAO	Adapter
1093	AGT	stimulus
1094	BMP-10	Ligand
1095	BMP-2	Ligand
1096	BMP-4	Ligand
1097	BMP-5	Ligand
1098	BMP-7	Ligand
1099	BMPR1	Receptor
1100	CCL11	chemokine
1101	CCNH	TF
1102	CSL	TF
1103	DKK2	SP
1104	EDG2	TF
1105	EDN1	Generics
1106	F5	Caspases
1107	FAF	Pro-Apoptic
1108	FCER1A	Receptor
1109	IL12	Pro-infammatory cytokines
1110	LAMR1	Receptor
1111	M-CSF	Generics
1112	MIP1A	Ligand
1113	MNK2	Kinase
1114	MYOD	TF
1115	NCOR	TF
1116	NICD	Generics
1117	NOGGIN	SP
1118	PROC	SP
1119	PROS1	SP
1120	SHH	SP
1121	T3R	TF
1122	TEP1	SP
1123	TGM2	SP
1124	VIP	Generics
1125	7TMR	Receptor
1126	AChE	synapse
1127	Acinus	ATPase
1128	ACK	GTPase inhibitor
1129	AIF	Transporter
1130	Akt	Kinase
1131	Alg-2	Ion-binding
1132	APIP	NA

1133	AR	TF
1134	ARAD	Receptor
1135	A-Raf	Kinase
1136	ARAI	Receptor
1137	ARHGEF6	GTPase activator
1138	ARP	TF
1139	ARTS	GTPase
1140	ASAP1	GTPase activator
1141	ASC	caspase activator
1142	ATF1	TF
1143	Aven	inhibition of caspase activation
1144	BCL10	regulation of apoptosis
1145	Bcl-b	A-A
1146	BCLG	regulation of apoptosis
1147	Bcl-rambo	regulation of apoptosis
1148	Bcl-w	A-A
1149	BCR	Kinase
1150	BFAR	apoptosis
1151	BI-1	regulation of apoptosis
1152	Bmf	regulation of apoptosis
1153	BNIP3	A-A
1154	BNIP3L	induction of apoptosis
1155	Bok	induction of apoptosis
1156	cAR	TF
1157	Cathepsin	lysosome
1158	Cby	TF
1159	CD19	Receptor
1160	CD28	Receptor
1161	CD3	Receptor
1162	CD47	NA
1163	Cer	cytokine
1164	CHOP10	TF
1165	CK19	cytoskeleton
1166	CKI	Kinase
1167	CL	NA
1168	Clk	Kinase
1169	Cn	NA
1170	CNG	channel
1171	cPLA2	TF
1172	CRAC	channel
1173	CryAB	NA
1174	CTLA-4	immune response
1175	CypA	isomerase
1176	DAP10	Adapter
1177	DAX-1	TF
1178	DcR3	Receptor
1179	DEDD1	TF
1180	DEFCAP	caspase activator
1181	Diversin	Kinase
1182	DJ1	NA
1183	DJ2	Ion-binding
1184	DLC2	microtubule
1185	DLK	Kinase
1186	Drp-1	hydrolase

1187	EDG4	Receptor
1188	eIF2B	translation initiation factor
1189	ERb	TF
1190	ErbB3	Receptor
1191	FAP-1	cytoskeleton
1192	FGF	GF
1193	FGFR	Receptor
1194	FLASH	TF
1195	FRP	NA
1196	FRS2	Receptor
1197	GAB	NA
1198	Gads	Adapter
1199	Gas	GTPase
1200	Gat2	symporter
1201	Gbg	peptidase
1202	Glycogen	transferase
1203	Graf	Rho GTPase activator
1204	Grb7	Adapter
1205	GRP1	Exchange factor
1206	HAX1	NA
1207	HBEGF	GF
1208	HINT1	hydrolase
1209	Hrk	regulation of apoptosis
1210	Hsp60	ATP-binding
1211	HSP72	ATP-binding
1212	HSPG	NA
1213	IBP	NA
1214	IBR	Receptor
1215	ICAT	TF
1216	Idax	NA
1217	IFNA	Cytokine
1218	IFNAR	Receptor
1219	IKKe	Kinase
1220	IL-13	chemokine
1221	IL13RA1	Receptor
1222	IL13RA2	Receptor
1223	IL2RG	Receptor
1224	IL4R	Receptor
1225	IL6R	Receptor
1226	ILPIP	Kinase
1227	I-mf	Receptor
1228	Ins	hormone
1229	IRAK4	Kinase
1230	IRAK-M	Kinase
1231	IRF3	TF
1232	ISGF3G	TF
1233	Itk	Kinae
1234	ITM2B	NA
1235	JAK3	Kinase
1236	JNK3	Kinase
1237	krm	NA
1238	Ku70	TF
1239	LFG	A-A
1240	Lgs	NA

1241	Livin	Ion-binding
1242	LPA	peptidase
1243	LRP	Receptor
1244	LZK	Receptor
1245	M3/6	Phosphatase
1246	MAP-1	apoptosis
1247	MAPKAP-K3	Kinase
1248	MCL1	regulation of apoptosis
1249	MEF2	TF
1250	MEK5	Kinase
1251	Mfn2	GTPase
1252	MKP2	Phosphatase
1253	MKP3	Phosphatase
1254	MKP4	Phosphatase
1255	MLK3	Kinase
1256	Mos	Kinase
1257	MSK2	Kinase
1258	MURR1	NA
1259	NDUFS1	Ion-binding
1260	NMT	transferase
1261	Noxa	NA
1262	Nrdp1	NA
1263	Nucling	NA
1264	N-WASP	NA
1265	Omi	NA
1266	p110Ia	Kinase
1267	p32	Receptor
1268	p38gamma	Kinase
1269	p55gamma	Kinase
1270	p68	transferase
1271	p85	regulation of apoptosis
1272	p85beta	Kinase
1273	PAC1	Kinase
1274	PACAP	Adapter
1275	PAG	immune response
1276	PAR1	Receptor
1277	PDE	NA
1278	PHKA2	Kinase
1279	PI3P	Adapter
1280	PIAS	TF
1281	PIM2	Kinase
1282	PKCz	Kinase
1283	PLC	Ion-binding
1284	PLCg-2	Ion-binding
1285	PLD	Ion-binding
1286	plectin	cytoskeleton
1287	PR-A	TF
1288	PrP	NA
1289	PTMA	TF
1290	PTP	Receptor
1291	Puma	apoptosis
1292	PYGB	phosphorylase
1293	Pygo	TF
1294	RasGRP	Ion_binding

1295	REA	TF
1296	RGS	TF
1297	RIP140	TF
1298	RLC	Receptor
1299	RNPK	Transporter
1300	RPTK	Kinase
1301	Ryk	Kinase
1302	SA	NA
1303	Ser-prot	Kinase
1304	SHARP	TF
1305	SHIP	Phosphatase
1306	SHP	TF
1307	SLC9A1	Transporter
1308	SLP-76	NA
1309	SMARCA4	TF
1310	SOCS	NA
1311	SPHK2	Kinase
1312	SRA1	TF
1313	STAT	transferase
1314	STAT6	TF
1315	TBK1	Kinase
1316	TCR	Receptor
1317	TEC	Kinase
1318	THRAP2	TF
1319	TLR1	Receptor
1320	TLR10	Receptor
1321	TLR5	Receptor
1322	TLR8	Receptor
1323	TOR	TF
1324	Tpl-2	Kinase
1325	TSAP6	electron transport
1326	Tsh	TF
1327	TUBB4Q	GTPase
1328	UBE1L	ligase
1329	UbiL	NA
1330	XAF1	Ion-binding
1331	ZAK	Kinase
1332	Zyxin	Ion-binding
1333	ACVRL1	Receptor
1334	ADAM10	Ion-binding
1335	ADAM17	Ion-binding
1336	AES	NA
1337	AIP4	ligase
1338	AML1	TF
1339	AML3	TF
1340	ANAPC2	Ligase
1341	AP2	Ion-binding
1342	AP2A1	SP
1343	AP2B1	Vesicle
1344	APP	Inhibitor
1345	APPL	NA
1346	APPL2	NA
1347	ARA160	TF
1348	ARA54	Ligase

1349	ARA55	Receptor
1350	ARA70	TF
1351	ARF4	GTPase
1352	ARNIP	Ion-binding
1353	ARRB2	NA
1354	ASCL1	TF
1355	ATF3	TF
1356	AXAM2	Peptidase
1357	AXIN2	Transducer
1358	BAG1	apoptosis
1359	BP75	TF
1360	BPAG1	Cytoskeleton
1361	BPAG2	SP
1362	CAMK	Transferase
1363	CAV2	NA
1364	CCNB2	NA
1365	CD151	Sensory
1366	CD44	Receptor
1367	CDC16	spindle
1368	CDC27	spindle
1369	CDC37	Kinase
1370	CDK9	Kinase
1371	CDX1	TF
1372	CEACAM1	NA
1373	CEBPB	TF
1374	CHK	transferase
1375	CIR	TF
1376	CITED1	TF
1377	CK2A2	Kinase
1378	CLCA1	Channel
1379	CLCA2	Channel
1380	CLTC	SP
1381	CNTN1	NA
1382	COPS5	NA
1383	CRI2	NA
1384	CRM1	Transporter
1385	CSF2RB	Receptor
1386	CSNK1D	Kinase
1387	CSNK1E	Kinase
1388	CTBP2	oxidoreductase
1389	CTCF	TF
1390	CTNND1	cytoskeleton
1391	DAAM1	NA
1392	DAB2	NA
1393	DCP1A	NA
1394	DD	Transporter
1395	DHH	Peptidase
1396	DLL1	Receptor
1397	DLL4	NA
1398	DSP	cytoskeleton
1399	DTX1	Ligase
1400	DVL2	NA
1401	DVL3	Kinase
1402	E2F5	TF

1403	E3B1	NA
1404	EBP1	GTPase
1405	EF1A	GTPase
1406	EIF3S2	NA
1407	ELK3	TF
1408	ELK4	TF
1409	ENG	NA
1410	EPPK1	cytoskeleton
1411	EPS15	Receptor
1412	EPS8	Adapter
1413	ERM	TF
1414	ERT	TF
1415	FES	Kinase
1416	FGR	Kinase
1417	FHL1	Ion-binding
1418	FHL2	Ion-binding
1419	FKBP12	isomerase
1420	FNTA	transferase
1421	FOXG1B	TF
1422	FOXH1	TF
1423	FURIN	Peptidase
1424	FZD2	Receptor
1425	FZD4	Receptor
1426	FZD5	Receptor
1427	FZD6	Receptor
1428	FZD7	Receptor
1429	FZD8	Receptor
1430	FZD9	Receptor
1431	FZR1	NA
1432	GAB2	SP
1433	GAS1	NA
1434	GATA1	TF
1435	GIPC	NA
1436	GJA1	Channel
1437	GP130	Receptor
1438	GRAP	Adapter
1439	GRB14	Adapter
1440	GRIM19	NA
1441	GRIP1	NA
1442	H3	NA
1443	HAT1	transferase
1444	HBO1	transferase
1445	HD	Transporter
1446	HERP1	TF
1447	HES1	TF
1448	HES5	TF
1449	HES6	TF
1450	HEY1	TF
1451	HGS	transporter
1452	HHIP	NA
1453	HIP1	cytoskeleton
1454	HIPK2	Transferse
1455	HIST3H3	nucleosome
1456	HIVEP3	Ion-binding

1457	HOXA9		TF
1458	HSPA8		ATPase
1459	ID1		TF
1460	ID2		TF
1461	ID3		TF
1462	ID4		TF
1463	IHH		Peptidase
1464	ITGA6		Receptor
1465	ITGB4		Receptor
1466	ITGB4BP		NA
1467	JAG1		SP
1468	JAG2		GF
1469	KIT		Receptor
1470	KPNB1		transporter
1471	KRT17		SP
1472	KRT18		SP
1473	KRT7		SP
1474	KRT8		SP
1475	LFNG		Transferase
1476	LIP1		NA
1477	LRP5		Receptor
1478	MAGEA1		NA
1479	MAGI3		Kinase
1480	MAML1		TF
1481	MAML2		TF
1482	MAML3		TF
1483	MCF2		cytoskeleton
1484	MFNG		Transferase
1485	MIG6		Rho GTPase activator
1486	Miz1		TF
1487	MMP7		matrilysin
1488	MST1R		Receptor
1489	MTA2		TF
1490	MUPP1		synaptosome
1491	MYB		TF
1492	MYF5		TF
1493	MYF6		TF
1494	MYOG		Tf
1495	NCK2		Adapter
1496	NOTCH2		A-A
1497	NOTCH3		Receptor
1498	NOTCH4		Receptor
1499	NOV		GF
1500	NSD1		TF
1501	NUMB		NA
1502	NUMBL		NA
1503	NUP153		Transporter
1504	NUP214		Transporter
1505		Oct-01	TF
1506		Oct-02	TF
1507	PAK6		Kinase
1508	PARD3		NA
1509	PATZ		TF
1510	PAX2		TF

1511	PAX5	TF
1512	PAX8	TF
1513	PEN2	NA
1514	PIAS3	TF
1515	PIAS4	TF
1516	PIK3C2B	Microsome
1517	PIK3CD	Kinase
1518	PITPN	Transporter
1519	PKN2	Kinase
1520	PLAGL1	Ion-binding
1521	PLCE1	Ion-binding
1522	PLSCR1	TF
1523	POFUT1	transferase
1524	POLR2A	TF
1525	PPP2CA	Translation regulation
1526	PPP2R2A	hydrolase
1527	PPP2R5B	hydrolase
1528	PRKAR1A	Kinase
1529	PRKAR1B	Kinase
1530	PRKCD	Kinase
1531	PRKCG	Kinase
1532	PRKCI	Kinase
1533	PSEN2	NA
1534	PSMD4	NA
1535	PTCH	Receptor
1536	PTCH2	Receptor
1537	PTK6	Kinase
1538	PTPN12	Phosphatase
1539	PTPRO	Receptor
1540	PTP-SL	Receptor
1541	RAB5	GTPase
1542	RAC3	GTPase
1543	Rad54l2	NA
1544	RALB	transporter
1545	RANBP9	NA
1546	RAP30	TF
1547	RAP74	Kinase
1548	RBBP7	NA
1549	REPS2	Ion-binding
1550	RGS16	GTPase
1551	RING1	TF
1552	RKIP	NA
1553	RNF4	TF
1554	RNTRE	GTPase
1555	ROR2	Kinase
1556	RTKN	NA
1557	RUVBL1	TF
1558	SALL1	TF
1559	SAP18	TF
1560	SAP30	TF
1561	SENP1	endopeptidase
1562	SF1	TF
1563	SFN	NA
1564	SH2D3C	Adapter

1565	SH3BGRL	adaptor
1566	SH3GL3	NA
1567	SHIP2	Phosphatase
1568	SHOC2	transferase
1569	SKI	NA
1570	SLC25A4	transporter
1571	SMURF1	ligase
1572	SMURF2	ligase
1573	SNIP1	NA
1574	SNRPD2	NA
1575	SNW1	spliceosome
1576	SNX1	Transporter
1577	SNX2	Transporter
1578	SNX4	Transporter
1579	SNX6	transporter
1580	SOCS1	NA

1581	SOCS3	A-A
1582	SOCS5	NA
1583	SOCS6	NA
1584	SOX1	TF
1585	SPDEF	TF
1586	SPRED1	NA
1587	SPRED2	NA
1588	SRY	TF
1589	STAMBPL1	NA
1590	STAP1	NA
1591	STAT5B	TF
1592	STK11	Kinase
1593	STK11IP	transferase
1594	STK36	TF
1595	STRAP	NA
1596	STUB1	ligase
1597	STXBP1	transporter
1598	SUMO2	NA
1599	SVIL	TF
1600	TBP	TF
1601	TBPIP	NA
1602	TCF12	TF
1603	TCF3	TF
1604	TCF4	TF
1605	TFDP2	TF
1606	TFIIH	TF
1607	TGFB2	GF
1608	TGFB3	GF
1609	TGFBRAP1	Receptor
1610	TGIF	TF
1611	TIEG2	TF
1612	TNIP1	NA
1613	TR4	TF
1614	UBC9	ligase
1615	UBE2D1	ligase
1616	UBE2D2	ligase
1617	UBE2D3	ligase
1618	UBE3A	ligase
1619	UXT	NA
1620	VANGL2	NA
1621	VAV3	Adaptor
1622	VDR	TF
1623	VIM	Transporter
1624	WDR12	NA
1625	WNT2	Matrix
1626	WNT3	Matrix
1627	WNT4	Matrix
1628	WNT5A	Matrix
1629	WNT7A	Matrix
1630	YAP1	NA
1631	YY1	TF
1632	ZFHX1B	TF
1633	ZFYVE9	Receptor
1634	ZNF259	Ion-binding
